# Robust Recovery of Optimally Smoothed Polymer Relaxation Spectrum from Stress Relaxation Test Measurements

**DOI:** 10.3390/polym16162300

**Published:** 2024-08-14

**Authors:** Anna Stankiewicz

**Affiliations:** Department of Technology Fundamentals, Faculty of Production Engineering, University of Life Sciences in Lublin, 20-612 Lublin, Poland; anna.m.stankiewicz@gmail.com

**Keywords:** viscoelasticity, relaxation time spectrum, linear relaxation modulus, optimally smoothed model, identification algorithm, model error, noise robustness

## Abstract

The relaxation spectrum is a fundamental viscoelastic characteristic from which other material functions used to describe the rheological properties of polymers can be determined. The spectrum is recovered from relaxation stress or oscillatory shear data. Since the problem of the relaxation spectrum identification is ill-posed, in the known methods, different mechanisms are built-in to obtain a smooth enough and noise-robust relaxation spectrum model. The regularization of the original problem and/or limit of the set of admissible solutions are the most commonly used remedies. Here, the problem of determining an optimally smoothed continuous relaxation time spectrum is directly stated and solved for the first time, assuming that discrete-time noise-corrupted measurements of a relaxation modulus obtained in the stress relaxation experiment are available for identification. The relaxation time spectrum model that reproduces the relaxation modulus measurements and is the best smoothed in the class of continuous square-integrable functions is sought. Based on the Hilbert projection theorem, the best-smoothed relaxation spectrum model is found to be described by a finite sum of specific exponential–hyperbolic basis functions. For noise-corrupted measurements, a quadratic with respect to the Lagrange multipliers term is introduced into the Lagrangian functional of the model’s best smoothing problem. As a result, a small model error of the relaxation modulus model is obtained, which increases the model’s robustness. The necessary and sufficient optimality conditions are derived whose unique solution yields a direct analytical formula of the best-smoothed relaxation spectrum model. The related model of the relaxation modulus is given. A computational identification algorithm using the singular value decomposition is presented, which can be easily implemented in any computing environment. The approximation error, model smoothness, noise robustness, and identifiability of the polymer real spectrum are studied analytically. It is demonstrated by numerical studies that the algorithm proposed can be successfully applied for the identification of one- and two-mode Gaussian-like relaxation spectra. The applicability of this approach to determining the Baumgaertel, Schausberger, and Winter spectrum is also examined, and it is shown that it is well approximated for higher frequencies and, in particular, in the neighborhood of the local maximum. However, the comparison of the asymptotic properties of the best-smoothed spectrum model and the BSW model a priori excludes a good approximation of the spectrum in the close neighborhood of zero-relaxation time.

## 1. Introduction

The relaxation spectrum is vital for constitutive models and for providing insight into the mechanical properties of polymers since, from the relaxation spectrum, other material functions used to describe rheological properties can be uniquely derived [1,2,3,4,5]. It is applied for description, analysis, and to accomplish the pre-assumed mechanical properties of different polymers [3,6,7,8].

The spectrum is not directly accessible via measurement and must be recovered from relaxation stress or oscillatory shear data. Numerous different methods have been proposed during the last seven decades for relaxation spectrum identification using data both from the stress relaxation experiment [6,9,10,11,12,13,14,15,16,17,18,19,20] and dynamic modulus tests [5,21,22,23,24,25,26,27,28,29,30,31]. The problem of relaxation spectrum identification is the ill-posed inverse problem of solving a system of Fredholm integral equations of the first kind obtained for discrete measurements of the relaxation modulus or the storage and loss modulus data. Therefore, the solutions, if any, are very sensitive to even small changes in the measurement data, which can lead to arbitrarily large changes in the determined relaxation spectrum. In consequence, robustly stable algorithms are required to solve it. The regularization of the original problem and/or constraining the set of admissible solutions is often necessary to construct such algorithms.

In the first works concerning the relaxation spectrum determination, the sets of the spectrum models were constrained to rather narrow classes of models. In 1948 Macey [9], while examining the viscoelastic properties of ceramic material, described the spectrum by the exponential–hyperbolic model, which corresponds to the modified Bessel function of the second kind and zero-order modeling the relaxation modulus. To describe the mechanical properties of polyisobutylene, Sips [10] introduced in 1950 a simple relaxation spectrum model given by the difference between two exponential functions, which implied a logarithmic model of the relaxation modulus. This model was next augmented to consider a long-term modulus by Yamamoto [11] and applied to study the rheological properties of the plant cell wall. The relaxation spectrum modeling in [9,10,11] is based on the known pairs of Laplace transforms.

The relaxation spectrum identification based on the Post–Widder formula [12] for the inverse Laplace transform was initiated by Alfrey and Doty [13], who proposed a simple differential model based on the first-order Post–Widder formula. Ter Haar [14] approximated the spectrum of relaxation frequencies using the modulus multiplied by time, the inverse of the relaxation frequency, which is, in fact, the Post–Widder inversion formula of the zero order. After many years, Bažant and Yunping [15] and Goangseup and Bažant [16] introduced a two-stage approach of approximating the stress relaxation data via multiple differentiable models of the relaxation modulus and, next, by applying the Post–Widder formula to designate the related model of the spectrum. The effectiveness of this approach depended, among other aspects, on the function applied to approximate the relaxation modulus. In [15], a logarithmic–exponential model of the relaxation modulus was proposed, for which the authors stated the third-order Post–Widder approximation to be satisfactory.

Both the algorithms based on the Post–Widder formula [13,14,15,16], using the least-squares approximation to guarantee the best fitting of the relaxation modulus measurement data, and those using the pairs of Laplace transforms [9,10,11], did not take into account the ill-posed nature of the relaxation spectrum determination problem.

Baumgaertel and Winter [21] used a nonlinear least-squares method for the recovery of a discrete relaxation time spectrum based on storage and loss modulus data, in which the number of discrete model modes was adjusted during the scheme iterations to avoid an ill-posedness of the problem and to enhance the model fit. Regularization was not applied here, as in several of the works discussed subsequently. Malkin [22] approximated a continuous relaxation spectrum using three constants: the maximum relaxation time, the slope in the logarithmic scale, and the form factor. Malkin et al. [23] derived a method of continuous relaxation spectrum calculations using the Mellin integral transform. The algorithm for the relaxation time spectrum approximation by power series was developed by Cho [24], which, using the regression of the dynamic modulus, provided a relaxation time spectrum as stable as the regularization method. The least squares identification without regularization was also applied by Babaei et al. [17] to determine the discrete Maxwell relaxation spectrum based on the stress relaxation data from the ramp test. Lv et al. [5] applied the extended least squares method (without regularization) to dynamic experiment data. Lee et al. [25] used the Chebyshev polynomials of the first kind to approximate dynamic moduli data and next derived a spectrum equation using the complex decomposition method and the Fuoss–Kirkwood relation without any regularization. Also, a derivative-based algorithm for continuous spectrum recovery, which is also appropriate for the experimental situation where oscillatory shear data are only available for a finite range of frequencies, as proposed by Anderssen et al. [26], does not use regularization.

Honerkamp and Weese [27,28] combined nonlinear regression with Tikhonov regularization and proposed a specific viscoelastic model described by the two-mode log-normal function. In turn, Davies and Goulding [29] approximated the relaxation spectrum by a sum of scaling kernel functions located at appropriately chosen points. In the Mustapha and Phillips algorithm [30], the sequence of nonlinear regularized least-squares problems, solved with respect to both the discrete relaxation times and the elastic moduli, was performed with an increasing number of discrete model modes. The approach proposed by Stadler and Bailly [31] is based on the relaxation spectrum approximation using a piecewise cubic Hermite spline with respective regularization. The regularized algorithms presented in [27,28,29,30,31] were developed for dynamic rheological tests. A methodology to calculate the relaxation spectrum of biopolymeric materials from stress relaxation data has been proposed by Kontogiorgos [32], combining Hansen’s least-squares numerical algorithm and Tikhonov regularization with the L-curve criterion chosen to select regularization parameter. Stankiewicz [18,19] and Stankiewicz et al. [20] derived different identification algorithms for the optimal regularized least-squares identification of relaxation time and frequency spectra in the classes of models defined by a finite series of different basis functions.

All the known methods for the relaxation spectrum optimal identification are based on the minimization of the quadratic model error defined directly for the measurements of the relaxation modulus or storage and loss modules. For example, in [5,17,21], the least-squares criterion was used directly, while in [18,19,20,21,27,28,30], regularized least-squares were used with various rules applied for the choice of regularization parameters to ensure the stability of the scheme and smoothness of the determined relaxation spectrum. In these papers, the mathematical formula describing the relaxation spectrum model was also, in advance, limited to the assumed class of admissible models.

In this paper, as a remedy for the ill-posed nature of the spectrum identification problem, the relaxation time spectrum model that reproduces the relaxation modulus measurements and which is the best smoothed in the class of continuous square-integrable functions was sought. This problem was formulated and solved in this paper for the first time for the spectrum of relaxation times. First, by applying the well-known Hilbert projection theorem, a new model was derived in which the best smoothing was achieved together with the simultaneous interpolation of relaxation modulus measurements. Next, to achieve noise robustness, the problem of the optimal smoothness of the spectrum model was augmented by introducing a quadratic term in the Lagrange functional of the original optimal spectrum smoothing problem. The necessary and sufficient optimality condition of the modified problem implied the best relaxation spectrum model as a finite sum of the basis functions given by the quotient of the exponential function and relaxation time. The components of the corresponding relaxation modulus model were given by simple hyperbolic functions. The permitted in advance small error of the relaxation modulus model combined with the specific modification of the Lagrange functional resulted in the model’s noise robustness. The complete computational procedure for determining the best-smoothed model was given. The singular value decomposition method was used for algebraic computations. Analytical formulas describing the relaxation modulus model error, the relaxation spectrum smoothness, and noise robustness indices were derived as quadratic positive definite forms dependent on the sampling instants applied in the experiment and the relaxation modulus measurements. The monotonicity of these indices was analyzed. The applicability of the proposed model and algorithm to determining the optimally smoothed models of polymers characterized by the short and middle relaxation times of the Gauss-like relaxation spectra and the long relaxation times of the Baumgaertel, Schausberger, and Winter spectrum was verified. The rough applicability analysis of the proposed approach to modeling the relaxation time spectra of different types, such as the Kohlrausch–Williams–Watts, fractional Maxwell, Scott–Blair, inverse power, and multiplied power–exponential laws, was also carried based on the compatibility of the boundary conditions of the real spectra and the best-smoothed model. These studies have shown the applicability of the new model and identification algorithm for the optimal recovery of the smoothed relaxation spectrum of polymers with a very wide range of relaxation times.

In summary, this paper addresses the ill-posed problem of identifying the relaxation time spectrum in a new, original way, previously unknown in the literature. In the known methods, different mechanisms are built in to obtain a smooth enough and noise-robust relaxation spectrum model. Here, a new approach is proposed where the optimally smoothed continuous square-integrable model of the relaxation spectrum, which reproduces relaxation modulus measurements with assumed acceptable small errors, is directly sought. This problem is mathematically formulated and solved, resulting in a unique, best-smoothed relaxation spectrum model and a complete identification algorithm. In the construction of known relaxation spectrum identification methods, the primary idea was the best model approximation, and the next one, implied by the ill-posed nature of the task, was the concept of smoothing the model by regularizing the original problem of the model optimal approximation. Here, the main measure of the model’s quality was the integral of the square of the relaxation spectrum, being simultaneously the measure of the model’s smoothness. The idea of the optimal model approximation is replaced here by the classical interpolation of measurement points. In the basic problem, precise interpolation is applied and is next modified to interpolation with a small error being allowed to ensure the noise robustness of the model and algorithm. The idea of the Tikhonov regularization technique results from the essence of the ill-posed problem—the lack of uniqueness for its solution or its discontinuity with respect to the measurement data. The problem of smoothing the relaxation spectrum posed here finds inspiration in the consequences of the ill-posed problem and sometimes catastrophic fluctuations of the obtained solution, and it eliminates these model-devastating effects.

In Appendix A, the proofs of the main results and derivations of some mathematical formulas are given. Some tables of numerical results were moved to Appendix B to increase the clarity of the article.

## 2. Materials and Methods

### 2.1. Relaxation Time Spectrum

The continuous relaxation time spectrum $H\left(\tau \right)$ of a linear viscoelastic material is defined by the following integral [1,26]:

(1) $$G\left(t\right)=\int_{0}^{\infty }\frac{H\left(\tau \right)}{\tau }e^{-t/\tau }d\tau ,$$

where $G\left(t\right)$ is the linear relaxation modulus at time t. The spectrum $H\left(\tau \right)$ is interpreted as a generalization of the discrete Maxwell spectrum to a continuous function [1,26] and characterizes the distributions of relaxation times τ.

### 2.2. Model of the Relaxation Spectrum

Assume that a model $H_{M}\left(\tau \right)$ of the relaxation spectrum $H\left(\tau \right)$ belongs to the space $L^{2}\left(0,\right.\left.\infty \right)$ of real-valued square-integrable functions on the interval $\left(0,\right.\left.\infty \right)$. Note that $L^{2}\left(0,\right.\left.\infty \right)$ is the Hilbert space with the norm $\left\|x\right\|=\sqrt{\langle x,x\rangle }$ induced by the inner product defined by the integral

$$\langle x,y\rangle =\int_{0}^{\infty }x\left(\tau \right)y\left(\tau \right)d\tau ,$$

where the functions $x\left(\tau \right),y\left(\tau \right)\in L^{2}\left(0,\right.\left.\infty \right)$ [33].

### 2.3. Identification

A classical way of conducting the identification experiment studying viscoelasticity is the stress-relaxation test, where time-dependent stress is studied for the step increase in the strain [1,34,35,36]. Suppose a certain stress relaxation experiment resulted in a set of measurements of the relaxation modulus $\left\{\bar{G}\left(t_{i}\right)=G\left(t_{i}\right)+z\left(t_{i}\right)\right\}$ at the sampling instants $t_{i}>0$, i=1,…,N, where $z\left(t_{i}\right)$ is the measurement noise. Identification, classically, consists of the selection, within the chosen class of models, of such a model that ensures the best fit to the measurement results. As a measure of the model’s accuracy, the quadratic index, used in the least squares approach, is usually taken. However, in this paper, we look, in the class of continuous square-integrable functions, for the best-smoothed model $H_{M}\left(\tau \right)$ that reproduces relaxation modulus measurements $\bar{G}\left(t_{i}\right)$. This problem is formulated and solved in the next section.

## 3. Results and Discussion

In this section, the problem of optimal smoothing of the relaxation spectrum model is mathematically formulated and solved using the Hilbert projection theorem. As a result, the best-smoothed relaxation spectrum model is derived in the form of a series of specific basis functions given by the quotient of the exponential function and the relaxation time. The respective model of the relaxation modulus was found to be described by a series of hyperbolic functions. The properties of these basis functions were examined, and the identifiable property of the best-smoothed model was demonstrated. For noise measurement data, the modification of the problem for the spectrum model’s smoothness was proposed by augmenting the Lagrange functional. A dual approach was applied to solve the modified problem, resulting in the necessary and sufficient optimality condition for the optimal relaxation spectrum model. Direct analytical formulas for the relaxation spectrum and modulus models are given; their numerical realization by the singular value decomposition of the basic matrix was proposed. The model smoothness, noise robustness for noisy measurements of the relaxation modulus, and the error of the relaxation modulus model were analyzed. A simple identification scheme was proposed. Finally, the results of simulation studies for polymers described by Gaussian-like and BSW relaxation spectra distributions are presented.

### 3.1. The Problem of Optimal Smoothing of the Relaxation Spectrum Model

Consider the following problem. Find function $H_{M}\left(\tau \right)\in L^{2}\left(0,\right.\left.\infty \right)$ that minimizes the integral square index:

(2) $${\left\|H_{M}\right\|}^{2}=\int_{0}^{\infty }H_{M}^{2}\left(\tau \right)d\tau \to \underset{H_{M}\left(\cdot \right)\;\in L^{2}\left(0,\right.\left.\infty \right)}{min},$$

under the constraints

(3) $$\bar{G}\left(t_{i}\right)=\int_{0}^{\infty }\frac{H_{M}\left(\tau \right)}{\tau }e^{-t_{i}/\tau }d\tau ,\;i=1,\ldots ,N.$$

Note, that the set of functions $H_{M}\left(\tau \right)\in L^{2}\left(0,\right.\left.\infty \right)$ satisfying linear constraints (3) is closed and convex. Since the Hilbert projection theorem [33] implies the existence of a unique element with a minimal norm in the nonempty closed and convex subset of the Hilbert space, the existence and uniqueness of the solution to the smoothing problem (2) and (3) follows.

The Lagrange functional of the optimization problem defined by (2) and (3) is as follows:

(4) $$L\left(H_{M},\lambda_{N}\right)=\int_{0}^{\infty }H_{M}^{2}\left(\tau \right)d\tau +\sum_{i=1}^{N}\lambda_{i}\left[\bar{G}\left(t_{i}\right)-\int_{0}^{\infty }\frac{H_{M}\left(\tau \right)}{\tau }e^{-t_{i}/\tau }d\tau \right],$$

where ${\lambda_{N}=\left[\lambda_{1},\ldots ,\lambda_{N}\right]}^{T}$ is a vector of Lagrange multipliers $\lambda_{i}$. The necessary and sufficient optimality conditions for the linear-quadratic optimization task (2) and (3) are given by the equation

(5) $$H_{M}\left(\tau \right)=\sum_{i=1}^{N}\lambda_{i}\frac{e^{-t_{i}/\tau }}{\tau },$$

together with the constraints of (3). Substituting $H_{M}\left(\tau \right)$, given by (5), into (3) yields the following system of equations:

(6) $$\bar{G}\left(t_{i}\right)=\int_{0}^{\infty }\sum_{j=1}^{N}\lambda_{j}\frac{e^{-t_{j}/\tau }}{\tau^{2}}e^{-t_{i}/\tau }d\tau =\sum_{j=1}^{N}\lambda_{j}\int_{0}^{\infty }\frac{1}{\tau^{2}}e^{-\left(t_{j}+t_{i}\right)/\tau }d\tau ,\;i=1,\ldots ,N.$$

By applying the substitution v=1/τ in the integrals of the right-hand side of (6) we obtain:

(7) $$\phi_{ij}=\int_{0}^{\infty }\frac{1}{\tau^{2}}e^{-\left(t_{j}+t_{i}\right)/\tau }d\tau =\int_{0}^{\infty }e^{-\left(t_{j}+t_{i}\right)v}dv=\frac{1}{t_{i}+t_{j}}.$$

Introducing the N×N dimensional symmetric matrix composed of the elements $\phi_{ij}$ in the i row and j column according to

(8) $$\Phi_{N}={\left[\phi_{ij}\right]}_{\begin{array}{c} i=1,\ldots ,N\\ j=1,\ldots ,\;N \end{array}}={\left[\frac{1}{t_{i}+t_{j}}\right]}_{\begin{array}{c} i=1,\ldots ,N\\ j=1,\ldots ,\;N \end{array}},$$

the system of Equation (6) can be rewritten in compact form as

(9) $${\bar{G}}_{N}=\Phi_{N}\lambda_{N},$$

with the vector of the relaxation modulus measurements

(10) $${\bar{G}}_{N}{=\left[\bar{G}\left(t_{1}\right),\ldots ,\bar{G}\left(t_{N}\right)\right]}^{T},$$

where superscript “*T*” indicates transpose. The main properties of the matrix $\Phi_{N}$ are summarized in the following proposition shown in Appendix A.1.

**Proposition 1.**  *For arbitrary sampling instants $t_{i}>0$, i=1,…,N, such that* $t_{i+1}>t_{i}$*, a symmetric* N×N  *matrix* $\Phi_{N}$  *defined by (7) and (8) is a positive definite Gram matrix, which can be expressed as*  $\Phi_{N}=\Phi_{N}^{1/2}\Phi_{N}^{1/2}$*, where*  $\Phi_{N}^{1/2}$  *is the unique symmetric non-singular positive definite square root of*  $\Phi_{N}$*. Then, the inverse matrix*  $\Phi_{N}^{-1}=\Phi_{N}^{-1/2}\Phi_{N}^{-1/2}$  *is a positive definite too.*

Since matrix $\Phi_{N}$ is non-singular, the unique solution of (9) is given by

(11) $$\lambda_{N}=\Phi_{N}^{-1}{\bar{G}}_{N}.$$

Therefore, by virtue of (5), the best-smoothed model is described by the finite series

(12) $${\bar{H}}_{N}\left(\tau \right)=\sum_{i=1}^{N}\lambda_{i}h_{i}\left(\tau \right)$$

of the basis functions (compare (5))

(13) $$h_{i}\left(\tau \right)=\frac{e^{-t_{i}/\tau }}{\tau },\;i=1,\;2,\ldots .$$

The lower index ‘N’ is used in ${\bar{H}}_{N}\left(\tau \right)$ to express the dependence on the number of measurements.

Introducing the notation

(14) $$h_{N}\left(\tau \right)={\left[h_{1}\left(\tau \right),\ldots ,h_{N}\left(\tau \right)\right]}^{T}={\left[\frac{e^{-t_{1}/\tau }}{\tau },\ldots ,\frac{e^{-t_{N}/\tau }}{\tau }\right]}^{T}$$

and bearing in mind (11), model (12) can be expressed in compact vector–matrix form as

(15) $${\bar{H}}_{N}\left(\tau \right)=\lambda_{N}^{T}h_{N}\left(\tau \right)={\bar{G}}_{N}^{T}\Phi_{N}^{-1}h_{N}\left(\tau \right).$$

In view of (1) and (12), the related model of the relaxation modulus is as follows:

$${\bar{G}}_{N}\left(t\right)=\int_{0}^{\infty }\frac{{\bar{H}}_{N}\left(\tau \right)}{\tau }e^{-t/\tau }d\tau =\int_{0}^{\infty }\sum_{i=1}^{N}\lambda_{i}\frac{h_{i}\left(\tau \right)}{\tau }e^{-t/\tau }d\tau$$

and can be described by the finite series

(16) $${\bar{G}}_{N}\left(t\right)=\sum_{i=1}^{N}\lambda_{i}\int_{0}^{\infty }\frac{e^{-\left(t_{i}+t\right)/\tau }}{\tau^{2}}d\tau =\sum_{i=1}^{N}\lambda_{i}\varphi_{i}\left(t\right)$$

of hyperbolic basis functions (compare (7))

(17) $$\varphi_{i}\left(t\right)=\frac{1}{t+t_{i}},\;i=1,\ldots ,N.$$

Similarly, using the right equality in (16) and Equation (11), we obtain

$${\bar{G}}_{N}\left(t\right)=\lambda_{N}^{T}\varphi_{N}\left(t\right)={\bar{G}}_{N}^{T}\Phi_{N}^{-1}\varphi_{N}\left(t\right),$$

where, in view of (17), the vector function $\varphi_{N}\left(t\right)$ is as follows:

(18) $$\varphi_{N}\left(t\right)={\left[\varphi_{1}\left(t\right),\ldots ,\varphi_{N}\left(t\right)\right]}^{T}={\left[\frac{1}{t+t_{1}},\ldots ,\frac{1}{t+t_{N}}\right]}^{T}.$$

The index

(19) $$I_{N}={\left\|H_{M}\left(\tau \right)\right\|}^{2}=\int_{0}^{\infty }H_{M}^{2}\left(\tau \right)d\tau$$

minimized in (2), is a direct measure of smoothing the relaxation spectrum model. For the optimal model ${\bar{H}}_{N}\left(\tau \right)$ (15), the smoothness index is as follows:

(20) $$I_{N}=\int_{0}^{\infty }{\bar{H}}_{N}^{2}\left(\tau \right)d\tau ={\bar{G}}_{N}^{T}\Phi_{N}^{-1}\int_{0}^{\infty }h_{N}\left(\tau \right)h_{N}^{T}\left(\tau \right)d\tau \;\Phi_{N}^{-1}{\bar{G}}_{N}.$$

Using (14), (7), and (8) we find

(21) $$\int_{0}^{\infty }h_{N}\left(\tau \right)h_{N}^{T}\left(\tau \right)d\tau =\Phi_{N}.$$

Thus, Equation (20) yields

(22) $$I_{N}={\bar{G}}_{N}^{T}\Phi_{N}^{-1}{\bar{G}}_{N},$$

which means that the smoothness of the optimal model depends both on the time instants $t_{i}$ selected for the stress relaxation experiment, affecting the matrix $\Phi_{N}$ and on the experiment results ${\bar{G}}_{N}$.

As a result, the following result can be stated.

**Theorem 1.**  *For arbitrary sampling instants $t_{i}>0$, i=1,…,N, such that* $t_{i+1}>t_{i},$ *the unique optimally smoothed model of the relaxation time spectrum defined by the optimization task (2) and (3) is given by ${\bar{H}}_{N}\left(\tau \right)={\bar{G}}_{N}^{T}\Phi_{N}^{-1}h_{N}\left(\tau \right)$, while the respective relaxation modulus model ${\bar{G}}_{N}\left(t\right)={\bar{G}}_{N}^{T}\Phi_{N}^{-1}\varphi_{N}\left(t\right)$ and the optimal smoothness index $I_{N}={\bar{G}}_{N}^{T}\Phi_{N}^{-1}{\bar{G}}_{N}$, where the vector functions $h_{N}\left(\tau \right)$ and $\varphi_{N}\left(t\right)$ are defined by (14) and (18), respectively, and matrix $\Phi_{N}$ is defined by (8) and (7).*

The relaxation spectrum ${\bar{H}}_{N}\left(\tau \right)$ that solves the optimization task (2) and (3), is the most smoothed model in the class of square-integrable functions that, simultaneously, guarantee the reconstruction of the measurements of the relaxation modulus. Some useful algebraic identities concerning the matrix $\Phi_{N}$ and vector $\varphi_{N}\left(t\right)$ are given in Appendix A.2.

### 3.2. Properties of the Basis Functions

The basis functions $h_{i}\left(\tau \right)$ (13) and $\varphi_{i}\left(t\right)$ (17) of the relaxation spectrum and modulus models are positive definite and depend on the times $t_{i}$ applied in the stress relaxation experiment. The greater the sampling instants $t_{i}$, the faster the basic functions $\varphi_{i}\left(t\right)$ decrease. By (13), the first derivative is as follows:

$$\frac{dh_{i}\left(\tau \right)}{d\tau }=\frac{t_{i}-\tau }{\tau^{3}}e^{-t_{i}/\tau }.$$

Thus, the basis functions $h_{i}\left(\tau \right)$ for ${\tau =t}_{i}$ have a global maximum equal to $h_{i}\left(\tau \right)=1/\left({et}_{i}\right)$, which decreases with increasing index i due to the assumed monotonicity of the sequence $\left\{t_{i}\right\}$. This means that increasing the number of measurements N, i.e., increasing the model components, can allow for the good modeling of multimodal spectra, which is confirmed by the second example presented in the final part of this paper.

Since for $\tau \to 0^{+}$, using the L’Hospital’s rule, we have

$$\underset{\tau \to 0^{+}}{lim}\;h_{i}\left(\tau \right)=\underset{\tau \to 0^{+}}{lim}\;\frac{\frac{1}{\tau }}{e^{t_{i}/\tau }}=\underset{\tau \to 0^{+}}{lim}\;\frac{-\frac{1}{\tau^{2}}}{-\frac{t_{i}}{\tau^{2}}e^{t_{i}/\tau }}=\underset{\tau \to 0^{+}}{lim}\;\frac{1}{t_{i}e^{t_{i}/\tau }}=0^{+},$$

and for τ→∞ the functions are $h_{i}\left(\tau \right)\to 0$, the best model ${\bar{H}}_{N}\left(\tau \right)$ tends to zero both for $\tau \to 0^{+}$ and τ→∞ (zero boundary conditions). The basis functions $\varphi_{i}\left(t\right)$ given by (17) monotonically decrease to zero as t→∞.

**Figure 1:**
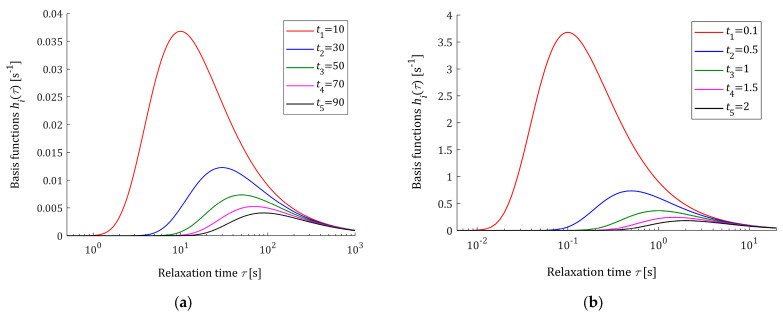
Basis functions $h_{i}\left(\tau \right)$ (13), i=1,…,5, of the relaxation spectrum model ${\bar{H}}_{N}\left(\tau \right)$ (12) for time sampling instants: (**a**) $t_{i}=10,\;30,\;50,\;70,\;90\;s$ and (**b**) $t_{i}=0.1,\;0.5,\;1,\;1.5,\;2\;s$.

**Figure 2:**
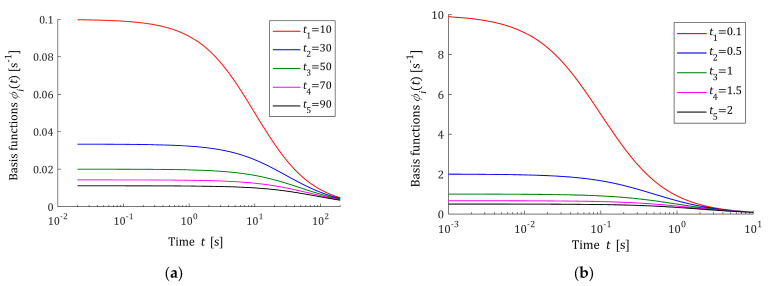
Basis functions $\varphi_{i}\left(t\right)$ (17), i=1,…,5, of the relaxation modulus model ${\bar{G}}_{N}\left(t\right)$ (16) for time sampling instants: (**a**) $t_{i}=10,\;30,\;50,\;70,\;90\;s$ and (**b**) $t_{i}=0.1,\;0.5,\;1,\;1.5,\;2\;s$.

The five first basis functions $h_{i}\left(\tau \right)$ (13) are shown in Figure 1 for the sampling instants $t_{i}=10,\;30,\;50,\;70,\;90$ and $t_{i}=0.1,\;0.5,\;1,\;1.5,\;2$ s. Figure 2 shows the related functions $\varphi_{i}\left(t\right)$ (17). The logarithmic scale is applied for the time axes in these figures. The basis functions $h_{i}\left(\tau \right)$ and $\varphi_{i}\left(t\right)$ are expressed in $s^{-1}$. From Figure 2, it can be seen that the monotonicity of basis functions $\varphi_{i}\left(t\right)$ is in good agreement with the courses of the relaxation modulus obtained in an experiment for real polymers; for example, these include elastic polyacrylamide hydrogels [35] (Figures 2a,b, 4a, A5, A7 and A8a), concrete [37] (Figure 13) and rubber [38] (Figure 2).

### 3.3. Identifiability

The basic and obvious requirement for any identification method is that if the real characteristic is described by a model from the considered class of models, and the measurements are noise-free, then the method should guarantee the unique determination of the real characteristic, i.e., ensure its identifiability [39,40].

Assume that the real spectrum is of the form

(23) $$H\left(\tau \right)=\sum_{j=1}^{N}a_{j}\frac{e^{-t_{j}/\tau }}{\tau },$$

where $a_{j}$ represents the real parameters. Introducing the vector $a={\left[a_{1},\ldots ,a_{N}\right]}^{T}$ and bearing in mind (13) and (14), spectrum (23) can be expressed as

(24) $$H\left(\tau \right)=a^{T}h_{N}\left(\tau \right).$$

Assume that the measurements of the relaxation modulus are noise-free. Thus, for i=1,…,N, by virtue of (1), (7) and (23), we have

$$\bar{G}\left(t_{i}\right)=G\left(t_{i}\right)=\int_{0}^{\infty }\sum_{j=1}^{N}a_{j}\frac{e^{-t_{j}/\tau }}{\tau^{2}}e^{-t_{i}/\tau }d\tau =\sum_{j=1}^{N}a_{j}\frac{1}{t_{j}+t_{i}}=\sum_{j=1}^{N}a_{j}\phi_{ij}.$$

Therefore, using (8) and (10), the vector of the relaxation modulus measurements can be expressed as

$${\bar{G}}_{N}={G_{N}=\Phi }_{N}a,$$

whence, according to (15) and (24), the relaxation spectrum model is as follows:

$${\bar{H}}_{N}\left(\tau \right)={\bar{G}}_{N}^{T}\Phi_{N}^{-1}h_{N}\left(\tau \right)=a^{T}\Phi_{N}\Phi_{N}^{-1}h_{N}=a^{T}h_{N}=H\left(\tau \right),$$

i.e., the model smoothing identification results in the determination of the real relaxation spectrum (23).

### 3.4. Modification

The value of the Lagrange multiplier $\lambda_{i}$ is the dual price [41], which in problem (2) and (3) is “paid” for satisfying the i-th constraint, i=1,…,N. The higher the value of $\lambda_{i}$ (precisely, the modulus of $\lambda_{i}$), the more difficult it is to meet this constraint and the stronger the chains it imposes. The impact of the fluctuations in the measurements of the relaxation modulus $\bar{G}\left(t_{i}\right)$, i.e., the impact of changes in the left side of each of Equation (3), on the smoothness of the spectrum is then greater. Really, by (22) and (11), we have

$$\frac{\partial I_{N}}{\partial {\bar{G}}_{N}}=2\Phi_{N}^{-1}{\bar{G}}_{N}=2{\bar{\lambda }}_{N}.$$

Therefore, the vector of the optimal Lagrange multipliers is the measure of the model’s smoothness index sensitivity with respect to the fluctuations of the relaxation modulus measurements. To reduce it, the value of the multiplier $\lambda_{i}$ should be reduced; precisely, the values of the modulus of $\lambda_{i}$ should be reduced.

The Lagrange functional of the optimization problem defined by (2) and (3) is described in Equation (4). In order to decrease the values of $\left|\lambda_{i}\right|$ and the functional (4), being maximized with respect to $\lambda_{i}$ according to the dual approach, is modified by introducing the quadratic term $\sum_{i=1}^{N}\lambda_{i}^{2}=\lambda_{N}^{T}\lambda_{N}$, which means that the modified Lagrange functional is defined as follows:

(25) $$L_{m}\left(H_{M},\lambda_{N}\right)=\int_{0}^{\infty }H_{M}^{2}\left(\tau \right)d\tau +\sum_{i=1}^{N}\lambda_{i}\left[\bar{G}\left(t_{i}\right)-\int_{0}^{\infty }\frac{H_{M}\left(\tau \right)}{\tau }e^{-t_{i}/\tau }d\tau \right]\;-\gamma \sum_{i=1}^{N}\lambda_{i}^{2},$$

where γ is a small positive constant and the weight that represents the relative importance of the square component $\lambda_{N}^{T}\lambda_{N}$ with respect to the original (non-modified) Lagrange functional $L\left(H_{M},\lambda_{N}\right)$ (4). The parameter γ has no physical interpretation as the regularization parameter in the classical Tikhonov regularization.

In Appendix A.3, the unique saddle point of the modified Lagrange functional (25) is found. The saddle point defines the modified best-smoothed model ${\bar{H}}_{N}^{\gamma }\left(\tau \right)$, which depends on the measurements $\left\{t_{i},\bar{G}\left(t_{i}\right)\right\}$ and the parameter γ introduced in (25).

**Theorem 2.**  *For arbitrary sampling instants*  $t_{i}>0$*,* i=1,…,N*, such that* $t_{i+1}>t_{i}$ *and the arbitrary non-negative parameter*  γ*, the model of the relaxation time spectrum*  ${\bar{H}}_{N}^{\gamma }\left(\tau \right)$  *defined by the unique saddle point of the modified Lagrange functional (25) is given by*

(26) $${\bar{H}}_{N}^{\gamma }\left(\tau \right)={\bar{G}}_{N}^{T}{\left(\Phi_{N}+4\gamma I_{N,N}\right)}^{-1}h_{N}\left(\tau \right),$$

*while the respective relaxation modulus model*

(27) $${\bar{G}}_{N}^{\gamma }\left(t\right)={\bar{G}}_{N}^{T}{\left(\Phi_{N}+4\gamma I_{N,N}\right)}^{-1}\varphi_{N}\left(t\right),$$

*where the vector functions*  $h_{N}\left(\tau \right)$  *and*  $\varphi_{N}\left(t\right)$  *are defined by (14) and (18), respectively, matrix*  $\Phi_{N}$  *is defined by (7) and (8) and* $I_{N,N}$  *is the*  N×N  *dimensional unit matrix.*

The upper index γ in the notations ${\bar{H}}_{N}^{\gamma }\left(\tau \right)$ and ${\bar{G}}_{N}^{\gamma }\left(t\right)$ indicates the dependence on the parameter γ introduced in the modified Lagrange functional (25).

To achieve the dimensional homogeneity of the components of the Lagrange functional (25), the multipliers $\lambda_{i}$ are expressed in Pa·s, while the unit of the parameter γ is $s^{-1}$. The dimensional homogeneity of the matrix $\Phi_{N}+4\gamma I_{N,N}$ is then achieved.

It is demonstrated in Appendix A.4 that for the optimal vectors of the Lagrange multipliers, $\lambda_{N}$ in (11) for original optimization task (2) and (4) and the vector $\lambda_{N}^{\gamma }$ (A13) of the saddle point of the modified Lagrange functional (25), the following inequality holds:

(28) $${\left(\lambda_{N}^{\gamma }\right)}^{T}\lambda_{N}^{\gamma }<\lambda_{N}^{T}\lambda_{N},$$

which means that $\left\|\lambda_{N}^{\gamma }\right\|<\left\|\lambda_{N}\right\|$, i.e., the purpose of the modification introduced into the Lagrange functional at the beginning of this section was achieved.

### 3.5. Model Error

Let us introduce, by analogy to the measurements vector ${\bar{G}}_{N}$ (10), the vector of the values of the relaxation modulus model (27) for all sampling instants $t_{i}$:

$${\bar{G}}_{N}^{\gamma }{=\left[{\bar{G}}_{N}^{\gamma }\left(t_{1}\right),\ldots ,{\bar{G}}_{N}^{\gamma }\left(t_{N}\right)\right]}^{T}.$$

For any $t=t_{i}$, by virtue of (27),

$${\bar{G}}_{N}^{\gamma }\left(t_{i}\right)={\bar{G}}_{N}^{T}{\left(\Phi_{N}+4\gamma I_{N,N}\right)}^{-1}\varphi_{N}\left(t_{i}\right)=\varphi_{N}^{T}\left(t_{i}\right){\left(\Phi_{N}+4\gamma I_{N,N}\right)}^{-1}{\bar{G}}_{N},$$

whence, bearing in mind Equation (A4), we have

$${\bar{G}}_{N}^{\gamma }=\Phi_{N}{\left(\Phi_{N}+4\gamma I_{N,N}\right)}^{-1}{\bar{G}}_{N}.$$

Therefore, for the model parameterized by γ>0, the relaxation modulus equations in (3) are not satisfied and the error of these equations is as follows:

(29) $$\varepsilon_{N}={\bar{G}}_{N}-{\bar{G}}_{N}^{\gamma }={\bar{G}}_{N}-\Phi_{N}{\left(\Phi_{N}+4\gamma I_{N,N}\right)}^{-1}{\bar{G}}_{N}.$$

Through the identity (A6), the model error $\varepsilon_{N}$ can be expressed as

(30) $$\varepsilon_{N}=4\gamma {\left(\Phi_{N}+4\gamma I_{N,N}\right)}^{-1}{\bar{G}}_{N},$$

and, bearing in mind (A13), can be equivalently expressed as $\varepsilon_{N}=2\gamma \lambda_{N}^{\gamma }$. Therefore, for γ=0 the model error $\varepsilon_{N}=0_{N}$, which is clear since in the original task (2) and (3) the constraints in Equation (3) are exactly satisfied; here, $0_{N}$ denotes the N dimensional vector of zero elements.

By (30), the square model error is given as follows:

(31) $$\varepsilon_{N}^{T}\varepsilon_{N}=16\gamma^{2}{\bar{G}}_{N}^{T}{\left(\Phi_{N}+4\gamma I_{N,N}\right)}^{-2}{\bar{G}}_{N}.$$

In Appendix A.5, the following result is proved.

**Proposition 2.**  *For arbitrary sampling instants*  $t_{i}>0$*,*  i=1,…,N*, such that* $t_{i+1}>t_{i},$ *and the arbitrary non-negative parameter*  γ*, the square model error*  $\varepsilon_{N}^{T}\varepsilon_{N}$ *(31) of the relaxation modulus equations monotonically increases as the function of the parameter*  γ *, which is strictly convex for*  γ  *such that*  $\Phi_{N}\geq 8\gamma I_{N,N},$  *and strictly concave in the case*  $\Phi_{N}\leq 8\gamma I_{N,N}$*.*

### 3.6. Smoothness

For model ${\bar{H}}_{N}^{\gamma }\left(\tau \right)$ (26), the smoothness index $I_{N}$ (19) is as follows:

$$I_{N}^{\gamma }=\int_{0}^{\infty }{\left[{\bar{H}}_{N}^{\gamma }\left(\tau \right)\right]}^{2}d\tau =\;{\bar{G}}_{N}^{T}{\left(\Phi_{N}+4\gamma I_{N,N}\right)}^{-1}\int_{0}^{\infty }h_{N}\left(\tau \right)h_{N}^{T}\left(\tau \right)d\tau \;{\left(\Phi_{N}+4\gamma I_{N,N}\right)}^{-1}{\bar{G}}_{N},$$

and, bearing in mind (21), can be expressed as

(32) $$I_{N}^{\gamma }={\bar{G}}_{N}^{T}{\left(\Phi_{N}+4\gamma I_{N,N}\right)}^{-1}\Phi_{N}{\left(\Phi_{N}+4\gamma I_{N,N}\right)}^{-1}{\bar{G}}_{N},$$

or, by applying identity (A7), an equivalent form useful for further differential analysis can be obtained

(33) $$I_{N}^{\gamma }={\bar{G}}_{N}^{T}\Phi_{N}^{1/2}{\left(\Phi_{N}+4\gamma I_{N,N}\right)}^{-2}\Phi_{N}^{1/2}{\bar{G}}_{N}.$$

Since, according to (22), the smoothness index for the original optimization task (2) and (3) can be expresses as $I_{N}={\bar{G}}_{N}^{T}\Phi_{N}^{1/2}\Phi_{N}^{-2}{\Phi_{N}^{1/2}\bar{G}}_{N}$, by inequality (A5), the next estimation follows:

$$I_{N}^{\gamma }<I_{N}$$

for any γ>0 and arbitrary measurement data, i.e., the smoothness of the spectrum model ${\bar{H}}_{N}^{\gamma }\left(\tau \right)$ (26) is stronger that the smoothness of the original model ${\bar{H}}_{N}\left(\tau \right)$ (15); this was the idea of the modification introduced in the Lagrange functional.

In Appendix A.6, the following formulas describing the first and second derivatives of $I_{N}^{\gamma }$ with respect to γ are derived:

(34) $$\frac{\partial }{\partial \gamma }I_{N}^{\gamma }=-8{\bar{G}}_{N}^{T}\Phi_{N}^{1/2}{\left(\Phi_{N}+4\gamma I_{N,N}\right)}^{-3}\Phi_{N}^{1/2}{\bar{G}}_{N},$$

and

(35) $$\frac{\partial^{2}}{\partial \gamma^{2}}I_{N}^{\gamma }=96\;{\bar{G}}_{N}^{T}\Phi_{N}^{1/2}{\left(\Phi_{N}+4\gamma I_{N,N}\right)}^{-4}\Phi_{N}^{1/2}{\bar{G}}_{N}.$$

Thus, the smoothness index $I_{N}^{\gamma }$ is the monotonically decreasing convex function of the parameter γ>0. The following rule holds: the greater the parameter γ is, the more highly bounded the fluctuations of the spectrum model ${\bar{H}}_{N}^{\gamma }\left(\tau \right)$ (26) are.

### 3.7. Noise Robustness

Following [18,19], as a reference point for the model ${\bar{H}}_{N}^{\gamma }\left(\tau \right)$ described by Equation (26), the model of the spectrum that can obtain for the same parameter γ, the same number of measurements N and the same time instants $t_{i}$ on the basis of ideal measurements of the relaxation modulus is considered, which is described as follows:

(36) $${\tilde{H}}_{N}^{\gamma }\left(\tau \right)=G_{N}^{T}{\left(\Phi_{N}+4\gamma I_{N,N}\right)}^{-1}h_{N}\left(\tau \right),$$

where $G_{N}$ is the vector of the noise-free relaxation modulus, i.e., $G_{N}={\left[\begin{array}{ccc} G\left(t_{1}\right) & \cdots & G\left(t_{N}\right) \end{array}\right]}^{T}$. In view of (26) and (36), we find

(37) $${\bar{H}}_{N}^{\gamma }\left(\tau \right)-{\tilde{H}}_{N}^{\gamma }\left(\tau \right)=z_{N}^{T}{\left(\Phi_{N}+4\gamma I_{N,N}\right)}^{-1}h_{N}\left(\tau \right),$$

where $z_{N}={\left[\begin{array}{ccc} z\left(t_{1}\right) & \cdots & z\left(t_{N}\right) \end{array}\right]}^{T}$ is the vector of measurement noises.

Consider the square integral index

(38) $$Q_{N}^{\gamma }=\int_{0}^{\infty }{\left[{\bar{H}}_{N}^{\gamma }\left(\tau \right)-{\tilde{H}}_{N}^{\gamma }\left(\tau \right)\right]}^{2}d\tau .$$

By (37), this index can be expressed as

$$Q_{N}^{\gamma }=z_{N}^{T}{\left(\Phi_{N}+4\gamma I_{N,N}\right)}^{-1}\int_{0}^{\infty }h_{N}\left(\tau \right)h_{N}^{T}\left(\tau \right)d\tau \;{\left(\Phi_{N}+4\gamma I_{N,N}\right)}^{-1}z_{N},$$

whence, by virtue of (21), we obtain

(39) $$Q_{N}^{\gamma }=z_{N}^{T}{\left(\Phi_{N}+4\gamma I_{N,N}\right)}^{-1}\Phi_{N}{\left(\Phi_{N}+4\gamma I_{N,N}\right)}^{-1}z_{N},$$

which, using the Gram property of $\Phi_{N}$ and using the identity (A7), can by rewritten as follows:

(40) $$Q_{N}^{\gamma }=z_{N}^{T}\Phi_{N}^{1/2}{\left(\Phi_{N}+4\gamma I_{N,N}\right)}^{-2}\Phi_{N}^{1/2}z_{N}.$$

Therefore, the noise robustness depends on the parameter γ, the measurement noises and the sampling instants $t_{i}$ that uniquely determine the matrix $\Phi_{N}$. Since both models are continuous with respect to the relaxation time τ, by virtue of (40), for any non-negative γ, the spectrum ${\bar{H}}_{N}^{\gamma }\left(\tau \right)$ tends to the noise-free spectrum ${\tilde{H}}_{N}^{\gamma }\left(\tau \right)$ for each time τ linearly with respect to the norm $\left\|z_{N}\right\|$, as $\left\|z_{N}\right\|\to 0$, and the faster this is, the larger the parameter γ.

By (A5), we have

(41) $$Q_{N}^{\gamma }<Q_{N}^{0}=z_{N}^{T}\Phi_{N}^{-1}z_{N},$$

which means better noise robustness than for the original model ${\bar{H}}_{N}\left(\tau \right)$ (15) for any γ>0.

Similarly, as for the smoothness index $I_{N}^{\gamma }$ (compare indices (33) and (40)), the following formulas describing derivatives of $Q_{N}^{\gamma }$ with respect to γ were derived:

$$\frac{\partial }{\partial \gamma }Q_{N}^{\gamma }=-8z_{N}^{T}\Phi_{N}^{1/2}{\left(\Phi_{N}+4\gamma I_{N,N}\right)}^{-3}\Phi_{N}^{1/2}z_{N},$$

and

$$\frac{\partial^{2}}{\partial \gamma^{2}}Q_{N}^{\gamma }=96\;z_{N}^{T}\Phi_{N}^{1/2}{\left(\Phi_{N}+4\gamma I_{N,N}\right)}^{-4}\Phi_{N}^{1/2}z_{N},$$

which means that $Q_{N}^{\gamma }$ is the monotonically decreasing convex function of the parameter γ>0 that takes the maximal value equal to $Q_{N}^{0}$ (41) for γ=0.

### 3.8. Algebraic Background of the Computational Algorithm

The singular value decomposition (SVD, [42]) technique can be used in numerical computations in order to determine the inverse matrix ${\left(\Phi_{N}+4\gamma I_{N,N}\right)}^{-1}$ in (26). Let SVD of the N×N dimensional matrix $\Phi_{N}$ (8) take the following form [42]:

(42) $$\Phi_{N}=U_{N}\Sigma_{N}U_{N}^{T},$$

where $\Sigma_{N}=diag\left(\sigma_{1},\ldots ,\sigma_{N}\right)\epsilon R^{N,N}$ is the diagonal matrix containing the singular values $\sigma_{i}$ of the matrix $\Phi_{N}$ [42], and the matrix $U_{N}\in R^{N,N}$ is orthogonal. Thus,

(43) $${\left(\Phi_{N}+4\gamma I_{N,N}\right)}^{-1}=U_{N}{\left(\Sigma_{N}+4\gamma I_{N,N}\right)}^{-1}U_{N}^{T},$$

where the N×N diagonal matrix ${\left(\Sigma_{N}+4\gamma I_{N,N}\right)}^{-1}$ is as follows:

$${\left(\Sigma_{N}+4\gamma I_{N,N}\right)}^{-1}=diag\left(1/\left(\sigma_{1}+4\gamma \right),\ldots ,1/\left(\sigma_{N}+4\gamma \right)\right).$$

Therefore, the optimal spectrum model (26) can be described by

(44) $${\bar{H}}_{N}^{\gamma }\left(\tau \right)={\left({\bar{g}}_{N}^{\gamma }\right)}^{T}h_{N}\left(\tau \right),$$

while the respective model (27) of the relaxation modulus is as follows:

$${\bar{G}}_{N}^{\gamma }\left(t\right)={\left({\bar{g}}_{N}^{\gamma }\right)}^{T}\varphi_{N}\left(t\right),$$

where the vector of model parameters is

(45) $${\bar{g}}_{N}^{\gamma }=U_{N}{\left(\Sigma_{N}+4\gamma I_{N,N}\right)}^{-1}U_{N}^{T}{\bar{G}}_{N}.$$

Using (42) and (43), the smoothness index $I_{N}^{\gamma }$ (32) is expressed as

(46) $$I_{N}^{\gamma }={\bar{G}}_{N}^{T}U_{N}\Omega_{N}U_{N}^{T}{\bar{G}}_{N},$$

where the N×N diagonal matrix $\Omega_{N}={\left(\Sigma_{N}+4\gamma I_{N,N}\right)}^{-1}\Sigma_{N}{\left(\Sigma_{N}+4\gamma I_{N,N}\right)}^{-1}$ takes the form

$$\Omega_{N}=diag\left(\sigma_{1}/{\left(\sigma_{1}+4\gamma \right)}^{2},\ldots ,\sigma_{N}/{\left(\sigma_{N}+4\gamma \right)}^{2}\right).$$

Similarly, using (42) and (43), the noise robustness index $Q_{N}^{\gamma }$ (39) can be rewritten as

$$Q_{N}^{\gamma }=z_{N}^{T}U_{N}\Omega_{N}U_{N}^{T}z_{N}.$$

Combining (31) and (43), we obtain the formula describing the square model error

(47) $$\varepsilon_{N}^{T}\varepsilon_{N}=16\gamma^{2}{\bar{G}}_{N}^{T}U_{N}{\left(\Sigma_{N}+4\gamma I_{N,N}\right)}^{-2}{U_{N}^{T}\bar{G}}_{N}.$$

### 3.9. Algorithm

The determination of the best-smoothed model of the relaxation time spectrum involves the following steps:

1. Choose the parameter γ>0.
2. Perform the experiment (stress relaxation test [1,34,35,36]) and record the measurements $\bar{G}\left(t_{i}\right)$, i=1,…,N, of the relaxation modulus at times $t_{i}>0,$ such that $t_{i+1}>t_{i}$.
3. Compute the matrix $\Phi_{N}$ and next determine SVD (42).
4. Compute the vector of model parameters ${\bar{g}}_{N}^{\gamma }$ (45).
5. Determine the spectrum of relaxation times ${\bar{H}}_{N}^{\gamma }\left(\tau \right)$ according to (44).
6. Determine the square model error $\varepsilon_{N}^{T}\varepsilon_{N}$ according to (47) and the smoothness index $I_{N}^{\gamma }$ using Formula (46).
7. Check if the smoothness of the spectrum model ${\bar{H}}_{N}^{\gamma }\left(\tau \right)$ measured by $I_{N}^{\gamma }$ and the error of the relaxation modulus model ${\bar{G}}_{N}^{\gamma }\left(t\right)$ measured by $\varepsilon_{N}^{T}\varepsilon_{N}$ are, simultaneously, satisfactory. If not, increase the parameter γ and repeat the computations starting from step 4. If yes, accept the current ${\bar{H}}_{N}^{\gamma }\left(\tau \right)$ as the best-smoothed relaxation spectrum model.

Only the SVD of the matrix $\Phi_{N}$ of computational complexity $O\left(N^{3}\right)$ [42] is a space- and time-consuming task in the scheme. However, for given sampling points, the SVD must be computed only once in step 3. The matrix $\Phi_{N}$ does not depend on the relaxation modulus measurements $\bar{G}\left(t_{i}\right)$. Therefore, when the identification scheme is applied for successive samples of the same material, step 3 should not be repeated whenever the same time instants $t_{i}$ are kept in the experiment. This is because, using (44) and (45), we have

$${\bar{H}}_{N}^{\gamma }\left(\tau \right)={\bar{G}}_{N}^{T}\vartheta_{N}^{\gamma }\left(\tau \right),$$

where the vector function

$$\vartheta_{N}^{\gamma }\left(\tau \right)=U_{N}{\left(\Sigma_{N}+4\gamma I_{N,N}\right)}^{-1}U_{N}^{T}h_{N}\left(\tau \right),$$

depends only on the sampling points $t_{i}$ and does not depend on the relaxation modulus measurements $\bar{G}\left(t_{i}\right)$. Therefore, the function $\vartheta_{N}^{\gamma }\left(\tau \right)$ must be computed only once and used to determine the model ${\bar{H}}_{N}^{\gamma }\left(\tau \right)$ for many samples whenever the same $t_{i}$ is kept.

### 3.10. Numerical Studies

For numerical studies, it is assumed that the viscoelastic properties are described by the Gaussian-like distribution of the relaxation spectrum, which is used to represent the rheological properties of numerous polymers, e.g., polyacrylamide gels [35], poly(methyl methacrylate) [43], polyethylene [44] and carboxymethylcellulose (CMC) [45]. Also, the spectra of many biopolymers have a Gaussian nature, for example, cold gel-like emulsions stabilized with bovine gelatin [46], fresh egg-white-hydrocolloids [45], some (wheat, potato, corn, and banana) native starch gels [47], the xanthan gum water solution [45] and wood [48,49]. The Baumgaertel, Schausberger, and Winter (BSW) spectrum [50,51] used to describe the viscoelasticity of polydisperse polymer melts [24,25], polybutadiene (PBD) [52], polymethylmethacrylate (PMMA) [52] and polymer pelts [53] is also considered.

The best-smoothed spectra models, the values of the square model error $\varepsilon_{N}^{T}\varepsilon_{N}$ (31), where the error $\varepsilon_{N}$ is defined by (29), the smoothness index $I_{N}^{\gamma }=\int_{0}^{\infty }{\left[{\bar{H}}_{N}^{\gamma }\left(\tau \right)\right]}^{2}d\tau$ given by Formula (32), and the square noise robustness index $Q_{N}^{\gamma }$ (38) expressed by Equation (39) are examined for different number of the measurements N and different values of the parameter γ.

The “real” materials and the best-smoothed models were simulated in Matlab R2023b, The Mathworks, Inc., Natick, MA, USA. For the singular value decomposition procedure *svd* was applied. A normal distribution with zero mean value and variance $\sigma^{2}$ as well as the uniform distribution were applied for the random independent generation of additive measurement noises.

### 3.11. Example I

Considering the polymer whose relaxation spectrum is described by the uni-modal Gaussian-like distribution as follows:

(48) $$H\left(\tau \right)=\vartheta e^{-{\left(\frac{1}{\tau }-m\right)}^{2/q}}/\tau ,$$

and where the parameters are as follows [54,55]: ϑ=31,520 Pa·s, $m=0.0912\;s^{-1}$ and $q=3.25\times 10^{-3}\;s^{-2}$. The related relaxation modulus is desribed by the function [55]:

(49) $$G\left(t\right)=\frac{\sqrt{\pi q}}{2}\vartheta \;e^{\frac{1}{4}t^{2}q-mt}erfc\left(\frac{\frac{1}{2}tq-m}{\sqrt{q}}\right),$$

where the complementary error function $erfc\left(x\right)$ is given by [56]:

$$erfc\left(x\right)=\frac{2}{\sqrt{\pi \;}\;}\int_{x}^{\infty }e^{{-z}^{2}}dz.$$

In the experiment, N sampling instants $t_{i}$ were generated with the constant period in the time interval of $T=\left[0,\;200\right]$ seconds, selected on the basis of the modulus $G\left(t\right)$ (49) course.

#### 3.11.1. Noise-Free Measurements

**Figure 3:**
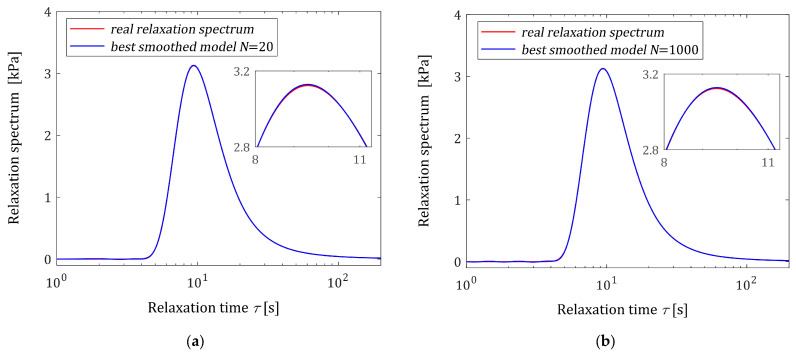
Relaxation time spectrum $H\left(\tau \right)$ (48) (solid red line) from Example I and the corresponding best-smoothed models ${\bar{H}}_{N}\left(\tau \right)$ (15) for N noise-free relaxation modulus measurements: (**a**) N=20; (**b**) N=1000.

**Figure 4:**
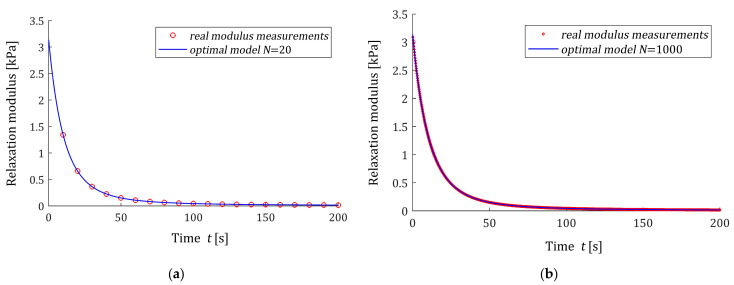
The measurements $\bar{G}\left(t_{i}\right)$ of the ‘real’ relaxation modulus $G\left(t\right)$ (49) (red circles) from Example I and the optimal model ${\bar{G}}_{N}\left(t\right)$ (16) for N noise-free relaxation modulus measurements: (**a**) N=20; (**b**) N=1000.

For noise-free measurements of the modulus $G\left(t\right)$ the best-smoothed model solving the original task (2) and (3) was determined for N=20, 100, 150, 200, 500, 1000 measurements. Two optimal models ${\bar{H}}_{N}\left(\tau \right)$ (15) and the ‘real’ spectrum $H\left(\tau \right)$ (48) are plotted in Figure 3. Small subfigures confirm the excellent model fit; real spectra described by the red lines practically coincide with the blue models both for the small and large number of measurements. This shows that in the case of noise-free measurements, the practically ideal approximation of the real relaxation spectrum was obtained even for a small number of measurements (N=20). In Figure 4, the related models of the relaxation modulus ${\bar{G}}_{N}\left(t\right)$ (16) are plotted; the measurements $\bar{G}\left(t_{i}\right)$ of the ‘real’ modulus $G\left(t\right)$ (49) are also marked. The values of the smoothness index $I_{N}$ (19) are given in Table 1.

#### 3.11.2. Noise-Corrupted Measurements

Additive independent measurement noises are generated by a normal distribution with zero mean value and variance $\sigma^{2}$. For the noise robustness analysis, the standard deviations σ=2,4,6 Pa were used. The parameters $\gamma =5\times 10^{-7},\;10^{-6},\;5\times 10^{-6},\;10^{-5}\;s^{-1}$ were applied.

**Figure 5:**
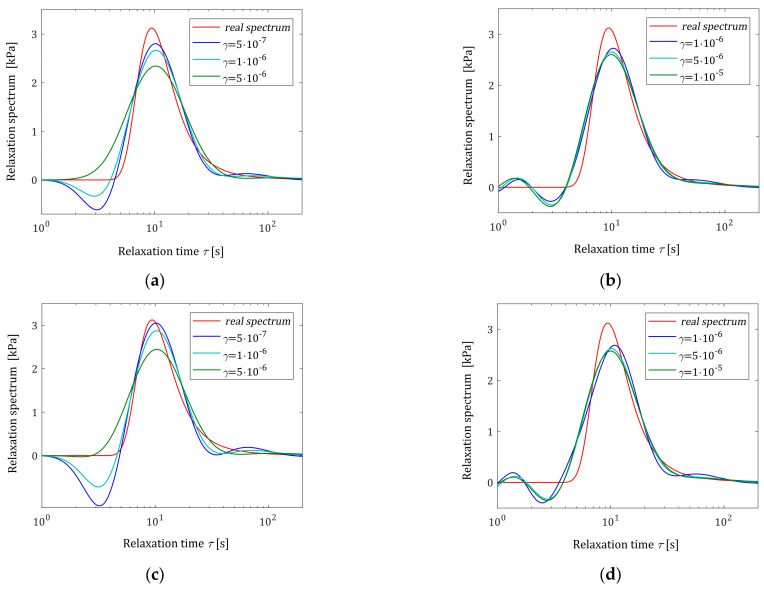

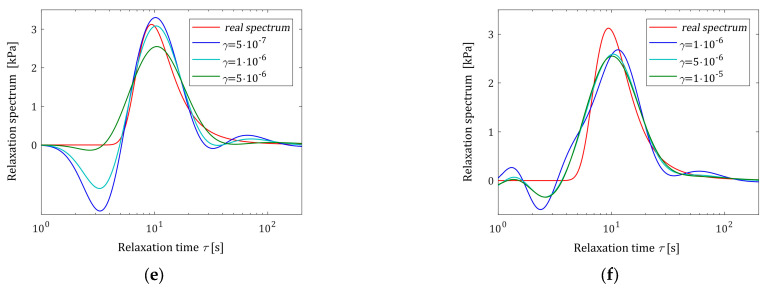
Relaxation time spectrum $H\left(\tau \right)$ (48) (solid red line) from Example I and the corresponding models ${\bar{H}}_{N}^{\gamma }\left(\tau \right)$ (26) for N measurements of the relaxation modulus corrupted by normally distributed additive independent noises with zero mean value and standard deviation σ: (**a**) σ=2 Pa and N=20; (**b**) σ=2 Pa and N=500; (**c**) σ=4 Pa and N=20; (**d**) σ=4 Pa and N=500; (**e**) σ=6 Pa and N=20; and (**f**) σ=6 Pa and N=500.

**Figure 6:**
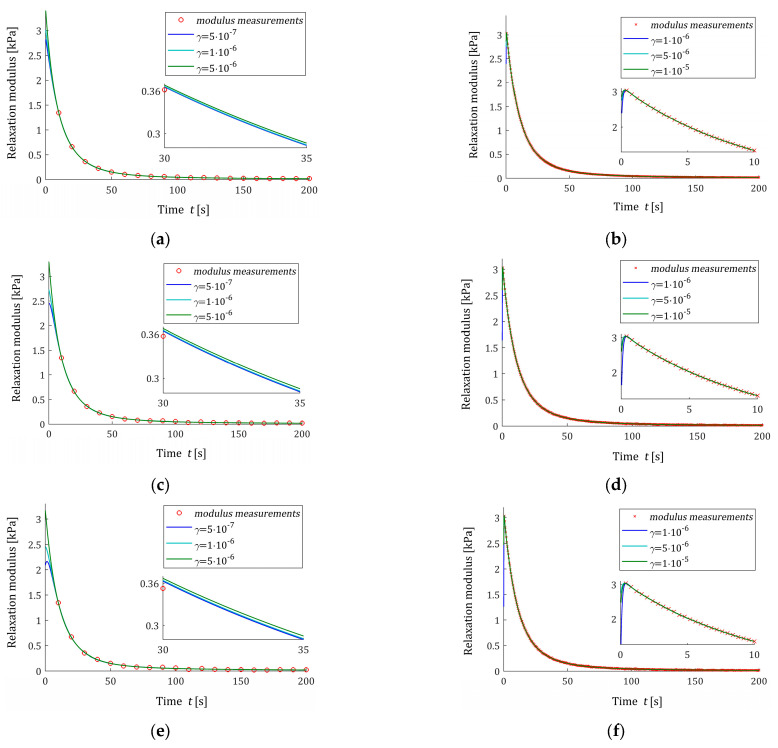
The measurements $\bar{G}\left(t_{i}\right)$ of the ‘real’ relaxation modulus $G\left(t\right)$ (49) (red circles) from Example I and the model ${\bar{G}}_{N}^{\gamma }\left(t\right)$ (27) for N measurements of the relaxation modulus corrupted by normally distributed additive independent noises with zero mean value and standard deviation σ: (**a**) σ=2 Pa and N=20; (**b**) σ=2 Pa and N=500; (**c**) σ=4 Pa and N=20; (**d**) σ=4 Pa and N=500; (**e**) σ=6 Pa and N=20; and (**f**) σ=6 Pa and N=500.

In Table 2, the values of the square model error $\varepsilon_{N}^{T}\varepsilon_{N}$ (31), the smoothness index $I_{N}^{\gamma }$ (32), and the square noise robustness index $Q_{N}^{\gamma }$ (38) are given for noises of σ=2Pa, while for the stronger noises, the same data are given in Table A1 and Table A2 in Appendix B. As previously, the exemplary courses of the spectrum models ${\bar{H}}_{N}^{\gamma }\left(\tau \right)$ (26) for N=20 and N=500 measurements are illustrated in Figure 5 for noises of σ=2, 4, 6 Pa, while the respective relaxation modulus models ${\bar{G}}_{N}^{\gamma }\left(t\right)$ (27) are depicted in Figure 6.

An inspection of Figure 5a,c,e shows that for each noise case, the number of N=20 measurements was not enough to obtain the satisfactory smoothness of the model ${\bar{H}}_{N}^{\gamma }\left(\tau \right)$ even for the weakest noises. So, N=20, which is good for noise-free case, fails here. However, for N=500 measurements, the model ${\bar{H}}_{N}^{\gamma }\left(\tau \right)$ is smoothed enough, and the influence of the regularization parameter γ is much weaker; see Figure 5b,d,f. Figure 6 and the values of the model errors $\varepsilon_{N}^{T}\varepsilon_{N}$ from Table 2, Table A1, and Table A2 confirm the excellent approximation of the relaxation modulus model, even though model imbalance is allowed.

The analytically shown monotonicity of the smoothness $I_{N}^{\gamma }$ and noise robustness $Q_{N}^{\gamma }$ indices, being the monotonically decreasing convex functions of the parameter γ, is reflected in the numerical studies. An inspection of the numerical results indicates that for N≥50 and any fixed parameter γ, the smoothness index $I_{N}^{\gamma }$ is a monotonically increasing function of the number of measurements; the slower this is, the larger the number N. An analysis of the asymptotic properties of the algorithm and optimal model ${\bar{H}}_{N}^{\gamma }\left(\tau \right)$ (26) as the number of measurements grows to infinity will be the subject of future studies.

### 3.12. Example II

**Figure 7:**
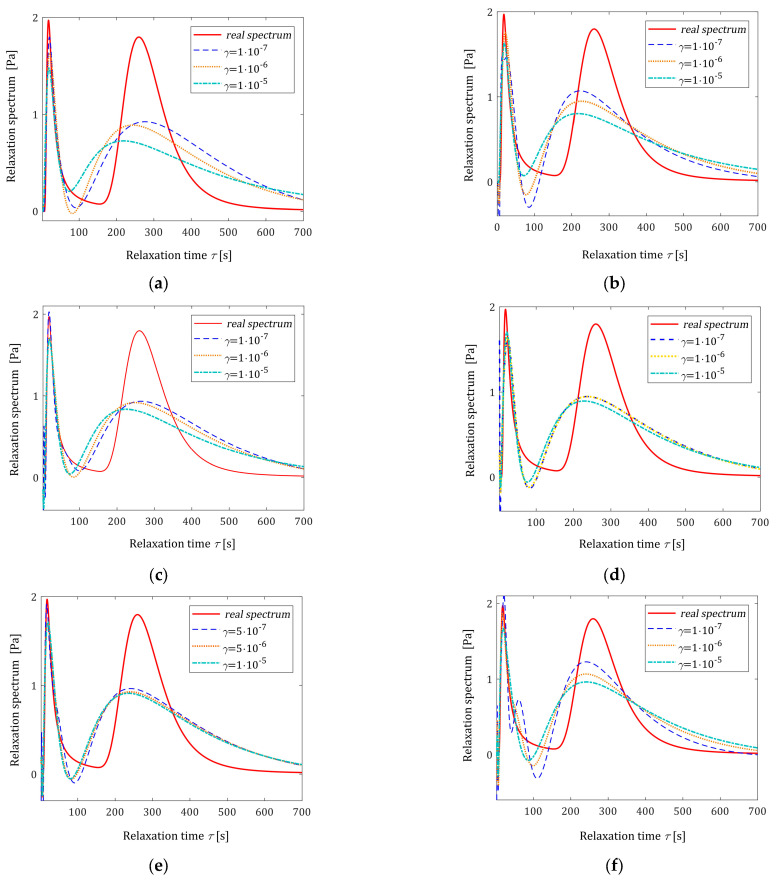
Relaxation time spectrum $H\left(\tau \right)$ (50) (solid red line) from Example II and the corresponding models ${\bar{H}}_{N}^{\gamma }\left(\tau \right)$ (26) determined for N measurements of the relaxation modulus corrupted by additive independent noises uniformly distributed on the interval $\left[-0.005,\;0.005\right]$ Pa: (**a**) N=50; (**b**) N=100; (**c**) N=200; (**d**) N=500; (**e**) N=1000; and (**f**) N=5000; the values of the parameter γ introduced in the modified Lagrange functional (25) are given in the figures.

**Figure 8:**
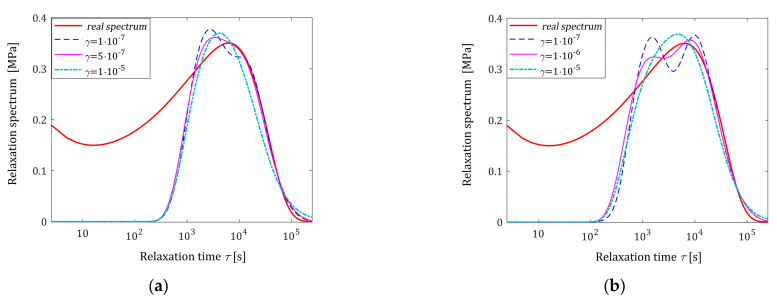

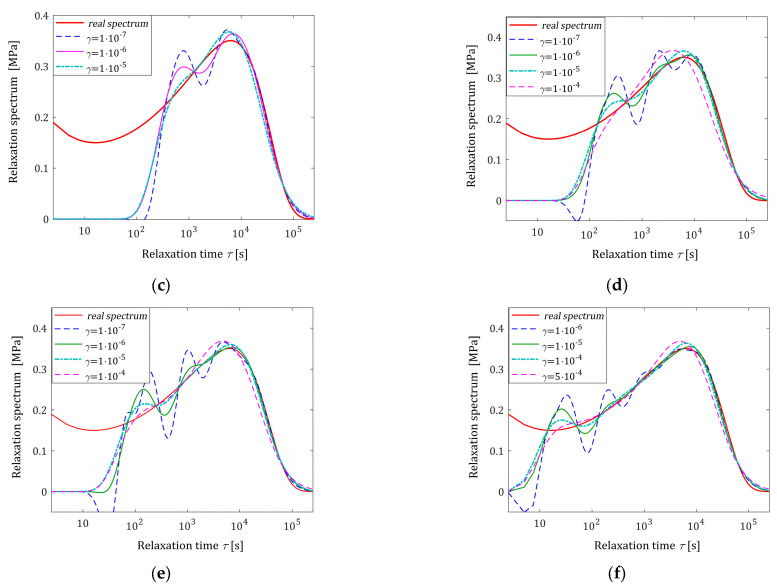
The relaxation time BSW spectrum (51) (solid red line) from Example III and the corresponding models ${\bar{H}}_{N}^{\gamma }\left(\tau \right)$ (26) for N measurements of the relaxation modulus corrupted by additive independent noises selected according to uniform distribution from the interval $\left[-0.005,\;0.005\right]\;MPa$ and parameters γ introduced in the modified Lagrange functional (25): (**a**) N=50; (**b**) N=100; (**c**) N=200; (**d**) N=500; (**e**) N=1000; and (**f**) N=5000.

Consider the double-mode Gaussian-like distribution of the relaxation spectrum [19,20,44]

(50) $$H\left(\tau \right)=\left[\beta_{1}e^{-{\left(\frac{1}{\tau }-m_{1}\right)}^{2/q_{1}}}+\beta_{2}e^{-{\left(\frac{1}{\tau }-m_{2}\right)}^{2/q_{2}}}\right]/\tau ,$$

where the parameters are as follows: $\beta_{1}=467\;Pa\cdot s$, $m_{1}=0.0037\;s^{-1}$, $q_{1}=1.124261\times 10^{-6}\;s^{-2}$, $\beta_{2}=39\;Pa\cdot s$, $m_{2}=0.045\;s^{-1}$ and $q_{2}=1.173\times 10^{-3}\;s^{-2}$. The double-Gaussian relaxation spectra are examined while developing new identification methods in [31] (Figure 2), [29] (Figures 9, 11 and 17), and [26] (Figures 2, 3, 6, 7–11 and 14). Such spectra describe the rheological properties of various polymers [44] (Figures 4b and 8b), polyacrylamide gels [35] (Figure A4), and wood [38]. The corresponding ‘real’ relaxation modulus is composed of two summands described by formulas like that of (49). In the experiment, N=50, 100, 200, 500, 1000, 5000 sampling instants $t_{i}$ were generated with the constant period in the time interval $T=\left[0,\;1550\right]$ seconds, selected in view of the course of the modulus. Following [19,20], additive measurement noises $z\left(t_{i}\right)$ were selected independently by random choice with uniform distribution on the interval $\left[-0.005,\;0.005\right]\;Pa$.

In Table 3, the values of the square model error $\varepsilon_{N}^{T}\varepsilon_{N}$ (31), the smoothness index $I_{N}^{\gamma }$ (32), and the square noise robustness index $Q_{N}^{\gamma }$ (38) are given. The spectrum models ${\bar{H}}_{N}^{\gamma }\left(\tau \right)$ (26) are illustrated in Figure 7 along with the real spectrum (50). Since, similar to the one-mode Gaussian relaxation spectrum, the relaxation modulus models ${\bar{G}}_{N}^{\gamma }\left(t\right)$ (27) for different N and γ values practically coincide, the respective figures are omitted here.

### 3.13. Example III

Consider the spectrum of relaxation times introduced by Baumgaertel, Schausberger, and Winter [50,51]

(51) $$H\left(\tau \right)=\left\{\beta_{1}{\left(\frac{\tau }{\tau_{c}}\right)}^{\rho_{1}}+\beta_{2}{\left(\frac{\tau }{\tau_{c}}\right)}^{\rho_{2}}\right\}e^{-\frac{\tau }{\tau_{max}}},$$

which is known to be effective in describing polydisperse polymer melts [24,25] with the parameters [25] $\beta_{1}=6.276\times 10^{-2}\;MPa$, $\beta_{2}=0.127\;MPa$, $\tau_{c}=2.481\;s$, $\tau_{max}=2.564\times 10^{4}\;s$, $\rho_{1}=0.25,$ and $\rho_{2}=-0.5$. As in [54], in the experiment, N time instants $t_{i}$ were sampled with the constant period in the time interval of $T=\left[0,10^{5}\right]$ seconds, where, following [18], the interval was selected in view of the course of the ‘real’ modulus $G\left(t\right)$ defined by (1). Additive measurement noises $z\left(t_{i}\right)$ were selected independently by random choice with uniform distribution on the interval $\left[-0.005,\;0.005\right]\;MPa$. The results of the numerical experiment are given in Table 4 and illustrated in Figure 8.

Since the real spectrum $H\left(\tau \right)$ (51) tends to infinity for τ→0 whenever at least one of the parameters $\rho_{1}$ and $\rho_{2}$ is negative, the best-smoothed model ${\bar{H}}_{N}^{\gamma }\left(\tau \right)$ (26) cannot adequately approximate this spectrum for small relaxation times τ; in the example for $0<\tau <10^{3}\;s$. This is well illustrated in Figure 8. However, this figure also shows that for a sufficiently large γ, the spectrum $H\left(\tau \right)$ for higher frequencies and its local maximum are well approximated.

### 3.14. Applicability for Identification of Relaxation Spectra of Different Types

The natural condition of this approach’s successful applicability follows from the properties of the best-smoothed model ${\bar{H}}_{N}^{\gamma }\left(\tau \right)$ (26) yielded by the properties of the basis functions $h_{i}\left(\tau \right)$ (13), which compose the vector $h_{N}\left(\tau \right)$ according to (14). Since for $\tau \to 0^{+}$ and τ→∞, the basis functions are $h_{i}\left(\tau \right)\to 0$ (c.f., Section 3.2), the best model ${\bar{H}}_{N}^{\gamma }\left(\tau \right)$ also tends to zero as the relaxation time τ tends to zero and to infinity. Therefore, zero boundary conditions limit the scope of applicability of the model and method to the real relaxation time spectra that satisfy these conditions. The example of the BSW spectrum demonstrates that the properties of the spectrum for $\tau \to 0^{+}$ are essential here since the real relaxation time spectra and the known spectra models tend to zero as the relaxation time τ tends to infinity.

The Kohlrausch–Williams–Watts (KWW) model of the stretched exponential relaxation described by [57]

(52) $$G\left(t\right)=G_{0}e^{-{\left(\frac{t}{\tau_{KWW}}\right)}^{\beta }},$$

where the stretching exponential 0<β<1, $\tau_{KWW}$ is the characteristic relaxation time and $G_{0}$ denotes the initial shear modulus, has been found by many researchers to be more appropriate than standard exponentials [57,58,59,60,61,62]. In spite of the simple, compact form of (52), the related unimodal [58] relaxation spectrum is described by the following infinite series [57,58]:

(53) $$H\left(\tau \right)=\frac{G_{0}}{\pi }\;\sum_{k=1}^{\infty }\frac{{\left(-1\right)}^{k+1}}{k!}\sin\left(\pi \beta k\right)\;\Gamma \left(\beta k+1\right)\;{\left(\frac{t}{\tau_{KWW}}\right)}^{\beta k},$$

which is based on Pollard’s representation of the stretched exponential as a Laplace integral [63], where $\Gamma \left(n\right)$ is Euler’s gamma function [64] (Equation (A.1.1)). However, for some specific stretching exponentials, namely $\beta =\frac{1}{2}$, $\beta =\frac{1}{3}$ and $\beta =\frac{2}{3}$, the KWW spectrum has a compact form described by some special functions [58]. For (53), both zero boundary conditions are satisfied; compare [57] (Figure 1a). Therefore, the proposed approach can be used to identify the spectrum of materials whose relaxation processes are described by the KWW model, e.g., polymer melts [59], the local segmental dynamics of poly(vinylacetate) [60], the segmental dynamics and the glass transition behavior of poly(2-vinylpyridine) [61], the relaxation of bone and bone collagen [62], alginate films while considering glycerol concentration [65], and even the relaxation processes of the onion structure in sine-oscillatory shear [66]. The best-smoothed model ${\bar{H}}_{N}^{\gamma }\left(\tau \right)$ (26) given by finite series may prove to be more useful than the original KWW spectrum (53).

In recent decades, non-integer order differential equations have increased interest in the modeling of time-dependent relaxation processes; the fractional Maxwell model (FMM) and the elementary Scott–Blair model are probably the most known rheological non-integer order models. The applicability of the FMM relaxation time spectrum, which is described by the compact analytical formula [54] (Equation (12)), to modeling the unimodal relaxation spectra of polymers was recently examined in [54]. However, it was demonstrated in [54] that the FMM relaxation spectrum tends to infinity as $\tau \to 0^{+}$ [54] (Proposition 2, Equation (19)); therefore, the exact fitting of the FMM-type spectrum by the proposed best-smoothed model in the whole relaxation time domain is excluded, which is similar for the BSW spectrum. The relaxation time spectrum of the Scott–Blair model described by the inverse power of the relaxation time with the non-integer exponent, see [54] (Equation (15)), also loses the zero boundary condition at zero relaxation time.

Similarly, real relaxation spectra which are well characterized by simple inverse power laws with various exponents [67]; for example, the power-type spectrum of cross-linking polymers at their gel point described by Winter and Chambon model with an exponent of −1/2 [68] and the spectrum of solution-polymerized styrene butadiene rubber described by a combined four-interval power model with fractional exponents [69] could not be successfully identified by the proposed method in the whole relaxation times domain. In turn, Winter’s power law relaxation time spectrum with a positive exponent [70,71] (Equation (2)), which was proposed to describe relaxation in many molecular and colloidal glasses, although satisfies the zero initial condition, could not be determined by the proposed algorithm due to its confined domain.

However, the proposed approach can be successfully applied to identify the relaxation spectra of materials such as bitumen, being characterized by the broadened power law model [71] (Equation (8)):

(54) $$H\left(\tau \right)=n_{\alpha }G_{c}{\left(\frac{t}{\tau_{\alpha }}\right)}^{n_{\alpha }}\;e^{-{\left(\frac{t}{\tau_{\alpha }}\right)}^{\beta }},$$

which multiplicative form combines the power law with an exponential of stretching parameter β. In the above model, the exponent $0<n_{\alpha }<1$, $\tau_{\alpha }$ is the longest relaxation time and $G_{c}$ is the plateau modulus. The unimodal spectrum (54) satisfies both zero boundary conditions; compare [71] (Figure 11a).

## 4. Conclusions

The objective of this paper was to develop a relaxation time spectrum model that could reproduce the relaxation modulus measurements and which is the best-smoothed in the class of continuous square-integrable functions. The unique optimal relaxation spectrum model was found to be described by a finite series of specific exponential–hyperbolic functions. A new identification algorithm was proposed in which the best smoothing of the model was achieved together with the simultaneous reconstruction of relaxation modulus measurements with small model errors. The analytical and numerical studies proved that using a developed model and algorithm, it is possible to determine the relaxation spectrum model for a wide class of polymers with zero boundary conditions, in particular, Gaussian-like distributed relaxation spectra. The model is smoothed and noise robust; small relaxation modulus model errors are obtained. The applicability of this approach to determining the Baumgaertel, Schausberger, and Winter spectrum was also examined, and it was proved that, due to the asymptotic properties of this spectrum, it can be well approximated for higher frequencies and, in particular, in the neighborhood of the local maximum. The rough applicability analysis, based on the consistency of the zero boundary conditions of the real spectra and the best-smoothed model, shows the possibility of using the proposed method and model to describe the relaxation spectra of different types that are characteristic of many polymers. However, the search for such a modification of the proposed approach so that it can also be applicable to the identification of spectra with non-zero boundary conditions for relaxation times approaching zero, like the BSW spectrum, will be the subject of future research. Generally, the properties of the method, including the smoothness of the relaxation spectrum, depend both on the experiment plan, i.e., on the sampling instants used in the relaxation test, and on the relaxation modulus measurements. The results of numerical studies confirm the analytically proved monotonic dependence on the gamma parameter: monotonically increasing for the square model error and monotonically decreasing for the noise robustness and spectrum model smoothness indices. However, the dependence of these indices on the number of measurements is not so clear and must be the subject of further studies.

Summarizing the numerical studies implies the following directions for future research:

- The asymptotic analysis of the model and identification algorithm properties as the number of measurements tends to infinity;
- The modification of the proposed approach for smoothing the spectrum model with a non-zero boundary condition for zero relaxation time;
- The modification for non-zero equilibrium modulus;
- The recurrent realization of the algorithm.

This method can be applied for any deformation process described by definitional Equation (1), i.e., both for uniaxial deformation, uniaxial stress, and uniaxial stretching, assuming that the relaxation modulus of the respective process is experimentally accessible. The relaxation time spectrum in the respective state (uniaxial deformation, stress, or stretching) is then determined. An appropriate modification of the algorithm can be developed to apply the concept of optimal relaxation spectrum model smoothing for oscillatory shear measurements of the storage and loss moduli. This will be the subject of further research.

## Figures and Tables

**Table 1:** The smoothness index $I_{N}$ (19) for noise-free N measurements of the relaxation modulus from Example I described by the relaxation spectrum $H\left(\tau \right)$ (48).

N	20	50	100	150	200	500	1000
$I_{N}\left[{kPa}^{2}\cdot s\right]$	70.937064	70.937063	70.937025	70.9370341	70.937296	70.9378314	70.937012

**Table 2:** For Example I, the square model error $\varepsilon_{N}^{T}\varepsilon_{N}$ (31), the smoothness index $I_{N}^{\gamma }=\int_{0}^{\infty }{\left[{\bar{H}}_{N}^{\gamma }\left(\tau \right)\right]}^{2}d\tau$, Equation (32), and the noise robustness index $Q_{N}^{\gamma }$ (38) for N measurements of the relaxation modulus corrupted by normally distributed additive independent noises with zero mean value and standard deviation σ=2 Pa; parameter γ is introduced in the modified Lagrange Functional (25).

γ $\left[s^{-1}\right]$	Index	N=20	N=50	N=100	N=500	N=1000
5 × 10^−7^	$\varepsilon_{N}^{T}\varepsilon_{N}$ $\left[{kPa}^{2}\right]$	1.39645 × 10^−4^	2.52753 × 10^−4^	4.62907 × 10^−4^	1.92494 × 10^−3^	3.88455 × 10^−3^
	$I_{N}^{\gamma }$ $\left[{kPa}^{2}\cdot s\right]$	72.99045	67.737749	68.723774	69.975844	73.348805
	$Q_{N}^{\gamma }$ $\left[{kPa}^{2}\cdot s\right]$	1.596146	1.662047	1.457413	1.371245	3.277104
1 × 10^−6^	$\varepsilon_{N}^{T}\varepsilon_{N}$ $\left[{kPa}^{2}\right]$	1.49648 × 10^−4^	2.57649 × 10^−4^	4.65545 × 10^−4^	1.92724 × 10^−3^	3.88936 × 10^−3^
	$I_{N}^{\gamma }$ $\left[{kPa}^{2}\cdot s\right]$	69.48318	65.994479	67.80256	69.162430	71.604073
	$Q_{N}^{\gamma }$ $\left[{kPa}^{2}\cdot s\right]$	0.891089	0.829375	0.757987	0.655363	1.298411
5 × 10^−6^	$\varepsilon_{N}^{T}\varepsilon_{N}$ $\left[{kPa}^{2}\right]$	2.08947 × 10^−4^	2.78699 × 10^−4^	4.85586 × 10^−4^	1.94192 × 10^−3^	3.90908 × 10^−3^
	$I_{N}^{\gamma }$ $\left[{kPa}^{2}\cdot s\right]$	63.29753	63.719666	65.798822	67.689507	69.559083
	$Q_{N}^{\gamma }$ $\left[{kPa}^{2}\cdot s\right]$	0.212948	0.293031	0.228734	0.103584	0.188545
1 × 10^−5^	$\varepsilon_{N}^{T}\varepsilon_{N}$ $\left[{kPa}^{2}\right]$	2.68258 × 10^−4^	3.03442 × 10^−4^	5.14355 × 10^−4^	1.96206 × 10^−3^	3.93448 × 10^−3^
	$I_{N}^{\gamma }$ $\left[{kPa}^{2}\cdot s\right]$	61.213971	62.869605	64.812065	66.994669	68.681175
	$Q_{N}^{\gamma }$ $\left[{kPa}^{2}\cdot s\right]$	0.102555	0.1849723	0.149022	5.95577 × 10^−2^	9.49178 × 10^−2^

**Table 3:** For the relaxation spectrum (50) from Example II described by double-mode Gaussian distribution: the square model error $\varepsilon_{N}^{T}\varepsilon_{N}$ (31), the smoothness index $I_{N}^{\gamma }$ (32), and the noise robustness index $Q_{N}^{\gamma }$ (38) for N measurements of the relaxation modulus corrupted by additive independent noises uniformly distributed on the interval $\left[-0.005,\;0.005\right]$ Pa; parameter γ introduced in the modified Lagrange functional (25).

γ $\left[s^{-1}\right]$	Index	N=50	N=100	N=200	N=500	N=1000	N=5000
1 × 10^−7^	$\varepsilon_{N}^{T}\varepsilon_{N}$ $\left[{Pa}^{2}\right]$	4.22629 × 10^−4^	8.08789× 10^−4^	1.62982 × 10^−3^	3.87154 × 10^−3^	7.89202 × 10^−3^	4.11897 × 10^−2^
	$I_{N}^{\gamma }$ $\left[{Pa}^{2}\cdot s\right]$	2.97202 × 10^2^	2.90993 × 10^2^	2.90362 × 10^2^	2.82977 × 10^2^	3.04036 × 10^2^	3.36177 × 10^2^
	$Q_{N}^{\gamma }$ $\left[{Pa}^{2}\cdot s\right]$	11.911735	10.130120	13.23408	15.429651	21.957947	19.365373
5 × 10^−7^	$\varepsilon_{N}^{T}\varepsilon_{N}$ $\left[{Pa}^{2}\right]$	4.49498 × 10^−4^	8.23669 × 10^−4^	1.64435 × 10^−3^	3.87762 × 10^−3^	7.91074 × 10^−2^	4.12242 × 10^−2^
	$I_{N}^{\gamma }$ $\left[{Pa}^{2}\cdot s\right]$	2.70677 × 10^2^	2.74519 × 10^2^	2.75150 × 10^2^	2.75893 × 10^2^	2.81813 × 10^2^	2.96481 × 10^2^
	$Q_{N}^{\gamma }$ $\left[{Pa}^{2}\cdot s\right]$	2.360449	1.926742	2.624791	2.092844	2.495385	2.836702
1 × 10^−6^	$\varepsilon_{N}^{T}\varepsilon_{N}$ $\left[{Pa}^{2}\right]$	4.90577 × 10^−4^	8.43919 × 10^−4^	1.66358 × 10^−3^	3.88467 × 10^−3^	7.92263 × 10^−2^	4.12478 × 10^−2^
	$I_{N}^{\gamma }$ $\left[{Pa}^{2}\cdot s\right]$	2.56575 × 10^2^	2.67642 × 10^2^	2.68545 × 10^2^	2.73497 × 10^2^	2.77623 × 10^2^	2.88112 × 10^2^
	$Q_{N}^{\gamma }$ $\left[{Pa}^{2}\cdot s\right]$	1.251268	0.969347	1.209452	0.880198	1.097522	1.369153
5 × 10^−6^	$\varepsilon_{N}^{T}\varepsilon_{N}$ $\left[{Pa}^{2}\right]$	8.80287 × 10^−4^	1.16953 × 10^−3^	1.90025 × 10^−3^	4.02082 × 10^−3^	8.03088 × 10^−2^	4.13474 × 10^−2^
	$I_{N}^{\gamma }$ $\left[{Pa}^{2}\cdot s\right]$	2.17932 × 10^2^	2.37719 × 10^2^	2.46356 × 10^2^	2.61489 × 10^2^	2.67451 × 10^2^	2.77109 × 10^2^
	$Q_{N}^{\gamma }$ $\left[{Pa}^{2}\cdot s\right]$	0.244248	0.220253	0.216093	0.169113	0.215385	0.248797
1 × 10^−5^	$\varepsilon_{N}^{T}\varepsilon_{N}$ $\left[{Pa}^{2}\right]$	1.37477 × 10^−3^	1.68512 × 10^−3^	2.29245 × 10^−3^	4.31822 × 10^−3^	8.23885 × 10^−2^	4.14497 × 10^−2^
	$I_{N}^{\gamma }$ $\left[{Pa}^{2}\cdot s\right]$	2.00761 × 10^2^	2.19869 × 10^2^	2.32877 × 10^2^	2.51385 × 10^2^	2.60383 × 10^2^	2.73583 × 10^2^
	$Q_{N}^{\gamma }$ $\left[{Pa}^{2}\cdot s\right]$	0.114042	0.113907	0.109599	0.090753	0.114361	0.119821

**Table 4:** For the polymer described by the BSW spectrum (51): the square model error $\varepsilon_{N}^{T}\varepsilon_{N}$ (31), the smoothness index $I_{N}^{\gamma }$ (32), and the noise robustness index $Q_{N}^{\gamma }$ (38) for N measurements of the relaxation modulus corrupted by additive independent noises selected according to uniform distribution from the interval $\left[-0.005,\;0.005\right]\;MPa$ and parameters γ introduced in the modified Lagrange functional (25).

γ $\left[s^{-1}\right]$	Index	N=50	N=100	N=200	N=500	N=1000
1 × 10^−7^	$\varepsilon_{N}^{T}\varepsilon_{N}$ $\left[{MPa}^{2}\right]$	4.50707 × 10^−4^	8.19820 × 10^−4^	1.65574 × 10^−3^	3.89542 × 10^−3^	7.93196 × 10^−3^
	$I_{N}^{\gamma }$ $\left[M{Pa}^{2}\cdot s\right]$	3.21654 × 10^3^	3.24851 × 10^3^	3.24718 × 10^3^	3.23537 × 10^3^	3.24043 × 10^3^
	$Q_{N}^{\gamma }$ $\left[M{Pa}^{2}\cdot s\right]$	11.891956	11.219052	10.864104	8.652512	10.942303
5 × 10^−7^	$\varepsilon_{N}^{T}\varepsilon_{N}$ $\left[{MPa}^{2}\right]$	5.16852 × 10^−4^	8.57969 × 10^−4^	1.67497 × 10^−3^	3.90799 × 10^−3^	7.94415 × 10^−3^
	$I_{N}^{\gamma }$ $\left[M{Pa}^{2}\cdot s\right]$	3.15685 × 10^3^	3.21397 × 10^3^	3.22838 × 10^3^	3.22318 × 10^3^	3.22606 × 10^3^
	$Q_{N}^{\gamma }$ $\left[M{Pa}^{2}\cdot s\right]$	2.175434	2.067436	2.0363525	2.006206	2.710421
1 × 10^−6^	$\varepsilon_{N}^{T}\varepsilon_{N}$ $\left[{MPa}^{2}\right]$	6.72858 × 10^−4^	9.49036 × 10^−4^	1.71894 × 10^−3^	3.93047 × 10^−3^	7.95659 × 10^−3^
	$I_{N}^{\gamma }$ $\left[M{Pa}^{2}\cdot s\right]$	3.10426 × 10^3^	3.18335 × 10^3^	3.213648 × 10^3^	3.21555 × 10^3^	3.22182 × 10^3^
	$Q_{N}^{\gamma }$ $\left[M{Pa}^{2}\cdot s\right]$	1.076912	0.975302	0.944682	0.997097	1.417322
5 × 10^−6^	$\varepsilon_{N}^{T}\varepsilon_{N}$ $\left[{MPa}^{2}\right]$	3.81118 × 10^−3^	2.95039 × 10^−3^	2.87847 × 10^−3^	4.44269 × 10^−3^	8.24008 × 10^−3^
	$I_{N}^{\gamma }$ $\left[M{Pa}^{2}\cdot s\right]$	2.82672 × 10^3^	3.00764 × 10^3^	3.11432 × 10^3^	3.17172 × 10^3^	3.19764 × 10^3^
	$Q_{N}^{\gamma }$ $\left[M{Pa}^{2}\cdot s\right]$	0.169205	0.230928	0.230358	0.204836	0.308728
1 × 10^−5^	$\varepsilon_{N}^{T}\varepsilon_{N}$ $\left[{MPa}^{2}\right]$	1.08157 × 10^−2^	7.46682 × 10^−3^	5.81259 × 10^−3^	5.84646 × 10^−3^	9.03681 × 10^−3^
	$I_{N}^{\gamma }$ $\left[M{Pa}^{2}\cdot s\right]$	2.58939 × 10^3^	2.85463 × 10^3^	3.01535 × 10^3^	3.12462 × 10^3^	3.17095 × 10^3^
	$Q_{N}^{\gamma }$ $\left[M{Pa}^{2}\cdot s\right]$	0.078727	0.114988	0.140981	0.118423	0.169938

## Data Availability

Data are contained within the article.

## Appendix A

### Appendix A.1. Proof of Proposition 1

Let us define the functions $h_{i}\left(v\right)=e^{-t_{i}v}$, i=1, 2,… and consider the function

(A1) $$f_{N}\left(v\right)=\sum_{i=1}^{N}g_{i}h_{i}\left(v\right),$$

where the real parameters $g_{i}$ compose the vector $g_{N}={\left[\begin{array}{ccc} g_{1} & \cdots & g_{N} \end{array}\right]}^{T}$. By (A1), for any vector $g_{K},$ we have

(A2) $$\int_{0}^{\infty }{\left[f_{N}\left(v\right)\right]}^{2}dv=\int_{0}^{\infty }{\left[\sum_{i=1}^{N}g_{i}h_{i}\left(v\right)\right]}^{2}dv,$$

which can be rewritten as

$$\int_{0}^{\infty }{\left[f_{N}\left(v\right)\right]}^{2}dv=\sum_{i=1}^{N}\sum_{j=1}^{N}g_{i}g_{j}\int_{0}^{\infty }h_{i}\left(v\right)h_{j}\left(v\right)dv,$$

and next, bearing in mind the right equality in (7), is expressed as

(A3) $$\int_{0}^{\infty }{\left[f_{N}\left(v\right)\right]}^{2}dv=\sum_{i=1}^{N}\sum_{j=1}^{N}g_{i}g_{j}\phi_{ij}.$$

Bearing in mind (8) and (A2), Equation (A3) is the quadratic form

$$\int_{0}^{\infty }{\left[f_{N}\left(v\right)\right]}^{2}dv=\int_{0}^{\infty }{\left[\sum_{i=1}^{N}g_{i}h_{i}\left(v\right)\right]}^{2}dv=g_{N}^{T}\Phi_{N}g_{N}.$$

Thus, $g_{N}^{T}\Phi_{N}g_{N}\geq 0$ for an arbitrary vector $g_{N}$, and $g_{N}^{T}\Phi_{N}g_{N}=0$, if and only if $\sum_{i=1}^{N}g_{i}h_{i}\left(v\right)=0$ for almost all v>0. Since the exponential functions $h_{1}\left(v\right)=e^{-t_{1}v},\ldots ,h_{N}\left(v\right)=e^{-t_{N}v}$, i.e., the kernel of the Laplace transformation, are linearly independent, the last equality holds if and only if $g_{i}=0$ for all i=1,…,N, i.e., only if the vector $g_{N}=0$, which yields the positive definiteness of $\Phi_{N}$. The Gram property of $\Phi_{N}$ follows directly from the definitional Formulas (7) and (8); its positive definiteness yields the existence of a unique symmetric positive definite square root matrix $\Phi_{N}^{1/2}$ in view of Theorem 7.2.6 in [72] concerning the positive semidefinite k-th roots of the Hermitian positive semidefinite matrices by lying k=2. Thus, $\Phi_{N}=\Phi_{N}^{1/2}\Phi_{N}^{1/2}$, and $\Phi_{N}^{1/2}={\left(\Phi_{N}^{1/2}\right)}^{T}$. By Theorem 4.2.1 in [42], the inverse matrix $\Phi_{N}^{-1}$ is a positive definite, too. The non-singularity of the square root $\Phi_{N}^{1/2}$ implies the inverse matrix formula $\Phi_{N}^{-1}=\Phi_{N}^{-1/2}\Phi_{N}^{-1/2}$; thus, this proposition is proved.

### Appendix A.2. Some Matrix Identities

In this appendix, some useful vector-matrix identities are given.

**Property A1.** *For arbitrary sampling instants $t_{i}>0$, i=1,…,N, such that* $t_{i+1}>t_{i}$ *, the matrix $\Phi_{N}$ (8) and vector function $\varphi_{N}\left(t\right)$, defined by (18), satisfy the equation*

(A4) $${{\left[\varphi_{N}\left(t_{1}\right),\ldots ,\varphi_{N}\left(t_{N}\right)\right]}^{T}=\left[\varphi_{N}\left(t_{1}\right),\ldots ,\varphi_{N}\left(t_{N}\right)\right]=\Phi }_{N}.$$

**Proof.** Equation (A4) follows directly from (8) and (18). □

**Property A2.** *For any*  γ>0  *and any positive definite symmetric matrix*  $\Phi_{N},$  *the following inequality*

(A5) $${\left(\Phi_{N}+4\gamma I_{N,N}\right)}^{-2}<\Phi_{N}^{-2},$$

*and equations*

(A6) $$I_{N,N}-\Phi_{N}{\left(\Phi_{N}+4\gamma I_{N,N}\right)}^{-1}=4\gamma {\left(\Phi_{N}+4\gamma I_{N,N}\right)}^{-1},$$

(A7) $${\left(\Phi_{N}+4\gamma I_{N,N}\right)}^{-1}\Phi_{N}^{1/2}=\Phi_{N}^{1/2}{\left(\Phi_{N}+4\gamma I_{N,N}\right)}^{-1}$$

*hold.*

**Proof.** Inequality (A5) results directly from the following inequality:

$$\Phi_{N}^{2}<\Phi_{N}^{2}+8\gamma \Phi_{N}+16\gamma^{2}I_{N,N}={\left(\Phi_{N}+4\gamma I_{N,N}\right)}^{2}.$$

The equality

$$I_{N,N}-\Phi_{N}{\left(\Phi_{N}+4\gamma I_{N,N}\right)}^{-1}=\left[\left(\Phi_{N}+4\gamma I_{N,N}\right)-\Phi_{N}\right]{\left(\Phi_{N}+4\gamma I_{N,N}\right)}^{-1}$$

implies identity (A6). For the Gram matrix $\Phi_{N}=\Phi_{N}^{1/2}\Phi_{N}^{1/2},$ we have

(A8) $$\Phi_{N}^{1/2}\left(\Phi_{N}+4\gamma I_{N,N}\right)=\left(\Phi_{N}+4\gamma I_{N,N}\right)\Phi_{N}^{1/2},$$

whence

$$\Phi_{N}=\Phi_{N}^{1/2}\Phi_{N}^{1/2}=\left(\Phi_{N}+4\gamma I_{N,N}\right)\Phi_{N}^{1/2}{\left(\Phi_{N}+4\gamma I_{N,N}\right)}^{-1}\Phi_{N}^{1/2},$$

and finally

(A9) $${\left(\Phi_{N}+4\gamma I_{N,N}\right)}^{-1}\Phi_{N}=\Phi_{N}^{1/2}{\left(\Phi_{N}+4\gamma I_{N,N}\right)}^{-1}\Phi_{N}^{1/2}.$$

From (A8), after multiplying by ${\left(\Phi_{N}+4\gamma I_{N,N}\right)}^{-1}$ on the right and on the left, we obtain identity (A7). □

### Appendix A.3. Proof of Theorem 2

A dual approach will be used to find the saddle point of the modified Lagrange Functional (25). First, the function $H_{M}\left(\tau \right)$ minimizing the Functional (25) is found, i.e., the primary optimization task

(A10) $$\underset{H_{M}\left(\cdot \right)\in L^{2}\left(0,\right.\left.\infty \right)}{min}L_{m}\left(H_{M},\lambda_{N}\right)=L_{m}\left({\bar{H}}_{M},\lambda_{N}\right),$$

is solved. Next, the dual function is maximized, and the vector of optimal Lagrange multipliers is found.

The necessary and sufficient optimality condition for the primary problem (A10) takes the form

$$2{\bar{H}}_{M}\left(\tau \right)-\sum_{i=1}^{N}\lambda_{i}\frac{e^{-t_{i}/\tau }}{\tau }=0,$$

from which we obtain

(A11) $${\bar{H}}_{M}\left(\tau \right)=\frac{1}{2}\sum_{i=1}^{N}\lambda_{i}\frac{e^{-t_{i}/\tau }}{\tau },$$

which, substituted into the modified Lagrange functional (25), yields the dual function

$$L_{D,m}\left(\lambda_{N}\right)=L_{m}\left({\bar{H}}_{M},\lambda_{N}\right),$$

given by

(A12) $$L_{D,m}\left(\lambda_{N}\right)=\sum_{i=1}^{N}\lambda_{i}\left[\bar{G}\left(t_{i}\right)-\frac{1}{4}\sum_{j=1}^{N}\lambda_{j}\int_{0}^{\infty }\frac{e^{-t_{j}/\tau }}{\tau }\frac{e^{-t_{i}/\tau }}{\tau }d\tau \right]-\gamma \sum_{i=1}^{N}\lambda_{i}^{2}.$$

By definition (7) and (8) of the matrix $\Phi_{N},$ the structure of the vectors ${\bar{G}}_{N}$ (10) and $\lambda_{N}$, the dual function (A12) can be expressed in compact form as

$$L_{D,m}\left(\lambda_{N}\right)=\lambda_{N}^{T}{\bar{G}}_{N}-\frac{1}{4}\lambda_{N}^{T}\Phi_{N}\lambda_{N}-\gamma \lambda_{N}^{T}\lambda_{N},$$

which, in view of Proposition 1, means that for any parameter γ>0 the dual function is strictly the concave function of $\lambda_{N}$ with the gradient

$$\frac{dL_{D,m}\left(\lambda_{N}\right)}{d\lambda_{N}}={\bar{G}}_{N}-\frac{1}{2}\Phi_{N}\lambda_{N}-2\gamma \lambda_{N}.$$

Therefore, the unique solution of the dual problem is given by the formula

(A13) $$\lambda_{N}^{\gamma }=2{\left(\Phi_{N}+4\gamma I_{N,N}\right)}^{-1}{\bar{G}}_{N}.$$

By (A11), and bearing in mind the notations (13) and (14), the corresponding relaxation spectrum model is expressed as follows:

(A14) $${\bar{H}}_{N}^{\gamma }\left(\tau \right)=\frac{1}{2}\sum_{i=1}^{N}\lambda_{i}^{\gamma }\frac{e^{-t_{i}/\tau }}{\tau }=\frac{1}{2}{\left(\lambda_{N}^{\gamma }\right)}^{T}h_{N}\left(\tau \right).$$

The pair $\left({\bar{H}}_{N}^{\gamma },\lambda_{N}^{\gamma }\right)$ is a unique saddle point of the Lagrange functional (25). The substitution of $\lambda_{N}^{\gamma }$ (A13) into (A14) yields Formula (26).

By (1) and the left equality in (A11), bearing in mind (16), for any t>0 we have

$${\bar{G}}_{N}^{\gamma }\left(t\right)=\int_{0}^{\infty }\frac{{\bar{H}}_{N}^{\gamma }\left(\tau \right)}{\tau }e^{-t/\tau }d\tau =\frac{1}{2}\sum_{i=1}^{N}\lambda_{i}^{\gamma }\int_{0}^{\infty }\frac{e^{-\left(t_{i}+t\right)/\tau }}{\tau^{2}}d\tau =\frac{1}{2}\sum_{i=1}^{N}\lambda_{i}^{\gamma }\varphi_{i}\left(t\right),$$

with the functions $\varphi_{i}\left(t\right)$ given by (17), which, through (18), can be expressed as

$${\bar{G}}_{N}^{\gamma }\left(t\right)=\frac{1}{2}{\left(\lambda_{N}^{\gamma }\right)}^{T}\varphi_{N}\left(t\right),$$

and, when combined with (A13), implies Formula (27). The result is proved.

### Appendix A.4. Derivation of the Inequality (28)

By (A13), we have

(A15) $${\left(\lambda_{N}^{\gamma }\right)}^{T}\lambda_{N}^{\gamma }=4{\bar{G}}_{N}^{T}{\left(\Phi_{N}+4\gamma I_{N,N}\right)}^{-2}{\bar{G}}_{N},$$

while for the Lagrange multiplier $\lambda_{N}$ (11) the respective value is

(A16) $$\lambda_{N}^{T}\lambda_{N}={\bar{G}}_{N}^{T}\Phi_{N}^{-2}{\bar{G}}_{N}.$$

Therefore, by virtue of (A15), (A16), and property (A5), for the optimal vectors of the Lagrange multipliers $\lambda_{N}$ (11) and $\lambda_{N}^{\gamma }$ (A13), the inequality (28) holds.

### Appendix A.5. Proof of Proposition 2

The following differential properties which hold for the arbitrary differentiable matrix functions $A\left(x\right)$ and $B\left(x\right)$ [42] (Equations (P2.1.2a) and (P2.1.2b)):

(A17) $$\frac{\partial }{\partial x}\left[A\left(x\right)B\left(x\right)\right]=\frac{\partial A\left(x\right)}{\partial x}B\left(x\right)+A\left(x\right)\frac{\partial B\left(x\right)}{\partial x},$$

(A18) $$\frac{\partial }{\partial x}\left[{A\left(x\right)}^{-1}\right]=-{A\left(x\right)}^{-1}\frac{\partial A\left(x\right)}{\partial x}{A\left(x\right)}^{-1},$$

assuming that matrix $A\left(x\right)$ is invertible, will be used.

By (A17), we obtain

(A19) $$\frac{\partial }{\partial x}{A\left(x\right)}^{-2}=\frac{\partial }{\partial x}\left[{A\left(x\right)}^{-1}{A\left(x\right)}^{-1}\right]=\frac{\partial {A\left(x\right)}^{-1}}{\partial x}{A\left(x\right)}^{-1}+{A\left(x\right)}^{-1}\frac{\partial {A\left(x\right)}^{-1}}{\partial x},$$

from which, including (A18), we immediately obtain the next useful differential formula:

(A20) $$\frac{\partial }{\partial x}{A\left(x\right)}^{-2}=-{A\left(x\right)}^{-1}\frac{\partial A\left(x\right)}{\partial x}{A\left(x\right)}^{-2}-{A\left(x\right)}^{-2}\frac{\partial A\left(x\right)}{\partial x}{A\left(x\right)}^{-1}.$$

From (31), it follows that

(A21) $$\frac{\partial }{\partial \gamma }\varepsilon_{N}^{T}\varepsilon_{N}=32\gamma {\bar{G}}_{N}^{T}{\left(\Phi_{N}+4\gamma I_{N,N}\right)}^{-2}{\bar{G}}_{N}+16\gamma^{2}{\bar{G}}_{N}^{T}{\frac{\partial }{\partial \gamma }\left(\Phi_{N}+4\gamma I_{N,N}\right)}^{-2}{\bar{G}}_{N}.$$

Since, by virtue of (A20),

$${\frac{\partial }{\partial \gamma }\left(\Phi_{N}+4\gamma I_{N,N}\right)}^{-2}=-8{\left(\Phi_{N}+4\gamma I_{N,N}\right)}^{-3},$$

Equation (A21) takes the form

$$\frac{\partial }{\partial \gamma }\varepsilon_{N}^{T}\varepsilon_{N}=32\gamma {\bar{G}}_{N}^{T}{\left(\Phi_{N}+4\gamma I_{N,N}\right)}^{-2}{\bar{G}}_{N}\;-16\cdot 8\cdot \gamma^{2}{\bar{G}}_{N}^{T}{\left(\Phi_{N}+4\gamma I_{N,N}\right)}^{-3}{\bar{G}}_{N},$$

and can be rewritten as follows

$$\frac{\partial }{\partial \gamma }\varepsilon_{N}^{T}\varepsilon_{N}=32\gamma {\bar{G}}_{N}^{T}{\left(\Phi_{N}+4\gamma I_{N,N}\right)}^{-3}\left[\left(\Phi_{N}+4\gamma I_{N,N}\right)-4\gamma I_{N,N}\right]{\bar{G}}_{N},$$

from which the next formula is directly obtained

(A22) $$\frac{\partial }{\partial \gamma }\varepsilon_{N}^{T}\varepsilon_{N}=32\gamma {\bar{G}}_{N}^{T}{\left(\Phi_{N}+4\gamma I_{N,N}\right)}^{-3}\Phi_{N}{\bar{G}}_{N}.$$

Therefore, using (A9), we obtain

$${\left(\Phi_{N}+4\gamma I_{N,N}\right)}^{-3}\Phi_{N}={\left(\Phi_{N}+4\gamma I_{N,N}\right)}^{-2}\Phi_{N}^{1/2}{\left(\Phi_{N}+4\gamma I_{N,N}\right)}^{-1}\Phi_{N}^{1/2},$$

whence, through the double use of identity (A7), we find

(A23) $$\begin{array}{c} {\left(\Phi_{N}+4\gamma I_{N,N}\right)}^{-3}\Phi_{N}=\\ {\left(\Phi_{N}+4\gamma I_{N,N}\right)}^{-1}{\left(\Phi_{N}+4\gamma I_{N,N}\right)}^{-1}\Phi_{N}^{1/2}{\left(\Phi_{N}+4\gamma I_{N,N}\right)}^{-1}\Phi_{N}^{1/2}=\\ {\left(\Phi_{N}+4\gamma I_{N,N}\right)}^{-1}\Phi_{N}^{1/2}{\left(\Phi_{N}+4\gamma I_{N,N}\right)}^{-2}\Phi_{N}^{1/2}=\\ \Phi_{N}^{1/2}{\left(\Phi_{N}+4\gamma I_{N,N}\right)}^{-3}\Phi_{N}^{1/2}. \end{array}$$

Combining (A22) and (A23) yields

(A24) $$\frac{\partial }{\partial \gamma }\varepsilon_{N}^{T}\varepsilon_{N}=32\gamma {\bar{G}}_{N}^{T}\Phi_{N}^{1/2}{\left(\Phi_{N}+4\gamma I_{N,N}\right)}^{-3}\Phi_{N}^{1/2}{\bar{G}}_{N},$$

from which the positive definiteness of the first derivative follows in view of the positive definiteness of the matrix ${\left(\Phi_{N}+4\gamma I_{N,N}\right)}^{-3}.$

From (A24), the second derivative is obtained

(A25) $$\frac{\partial^{2}}{\partial \gamma^{2}}\varepsilon_{N}^{T}\varepsilon_{N}=32{\bar{G}}_{N}^{T}\Phi_{N}^{1/2}{\left(\Phi_{N}+4\gamma I_{N,N}\right)}^{-3}\Phi_{N}^{1/2}{\bar{G}}_{N}\;+32\gamma {\bar{G}}_{N}^{T}\Phi_{N}^{1/2}\frac{\partial }{\partial \gamma }{\left(\Phi_{N}+4\gamma I_{N,N}\right)}^{-3}\Phi_{N}^{1/2}{\bar{G}}_{N}.$$

Applying (A17) again to the second term in (A25) gives

$$\frac{\partial^{2}}{\partial \gamma^{2}}\varepsilon_{N}^{T}\varepsilon_{N}=32{\bar{G}}_{N}^{T}\Phi_{N}^{1/2}{\left(\Phi_{N}+4\gamma I_{N,N}\right)}^{-3}\Phi_{N}^{1/2}{\bar{G}}_{N}\;+32\gamma {\bar{G}}_{N}^{T}\Phi_{N}^{1/2}\frac{\partial }{\partial \gamma }{\left(\Phi_{N}+4\gamma I_{N,N}\right)}^{-1}{\left(\Phi_{N}+4\gamma I_{N,N}\right)}^{-2}\Phi_{N}^{1/2}{\bar{G}}_{N}\;+32\gamma {\bar{G}}_{N}^{T}\Phi_{N}^{1/2}{\left(\Phi_{N}+4\gamma I_{N,N}\right)}^{-1}\frac{\partial }{\partial \gamma }{\left(\Phi_{N}+4\gamma I_{N,N}\right)}^{-2}\Phi_{N}^{1/2}{\bar{G}}_{N},$$

whence, using differential Formulas (A18) and (A20), we obtain

$$\frac{\partial^{2}}{\partial \gamma^{2}}\varepsilon_{N}^{T}\varepsilon_{N}=32{\bar{G}}_{N}^{T}\Phi_{N}^{1/2}{\left(\Phi_{N}+4\gamma I_{N,N}\right)}^{-3}\Phi_{N}^{1/2}{\bar{G}}_{N}\;-32\cdot 4\cdot \gamma {\bar{G}}_{N}^{T}\Phi_{N}^{1/2}{\left(\Phi_{N}+4\gamma I_{N,N}\right)}^{-2}{\left(\Phi_{N}+4\gamma I_{N,N}\right)}^{-2}\Phi_{N}^{1/2}{\bar{G}}_{N}\;-32\cdot 8\cdot \gamma {\bar{G}}_{N}^{T}\Phi_{N}^{1/2}{\left(\Phi_{N}+4\gamma I_{N,N}\right)}^{-1}{\left(\Phi_{N}+4\gamma I_{N,N}\right)}^{-3}\Phi_{N}^{1/2}{\bar{G}}_{N},$$

and next

$$\frac{\partial^{2}}{\partial \gamma^{2}}\varepsilon_{N}^{T}\varepsilon_{N}=32{\bar{G}}_{N}^{T}\Phi_{N}^{1/2}{\left(\Phi_{N}+4\gamma I_{N,N}\right)}^{-3}\Phi_{N}^{1/2}{\bar{G}}_{N}\;-32\cdot 12\cdot \gamma {\bar{G}}_{N}^{T}\Phi_{N}^{1/2}{\left(\Phi_{N}+4\gamma I_{N,N}\right)}^{-4}\Phi_{N}^{1/2}\Phi_{N}^{1/2}{\bar{G}}_{N},$$

which can be finally expressed as the quadratic form

$$\frac{\partial^{2}}{\partial \gamma^{2}}\varepsilon_{N}^{T}\varepsilon_{N}=\;32{\bar{G}}_{N}^{T}\Phi_{N}^{1/2}{\left(\Phi_{N}+4\gamma I_{N,N}\right)}^{-2}\left(\Phi_{N}-8\gamma I_{N,N}\right){\left(\Phi_{N}+4\gamma I_{N,N}\right)}^{-2}\Phi_{N}^{1/2}{\bar{G}}_{N}.$$

The matrix ${\left(\Phi_{N}+4\gamma I_{N,N}\right)}^{-2}$ is a positive definite. The conditions concerning convexity and concativity of the square model error $\varepsilon_{N}^{T}\varepsilon_{N}$ result directly from the conditions of positive or negative definiteness of the matrix $\left(\Phi_{N}-8\gamma I_{N,N}\right)$. The proposition is proved.

### Appendix A.6. Derivation of Formulas (34) and (35)

By (33) and differential Formulas (A19) and (A18), for any γ>0, we have

$$\frac{\partial }{\partial \gamma }I_{N}^{\gamma }=-4{\bar{G}}_{N}^{T}\Phi_{N}^{1/2}{\left(\Phi_{N}+4\gamma I_{N,N}\right)}^{-2}{\left(\Phi_{N}+4\gamma I_{N,N}\right)}^{-1}\Phi_{N}^{1/2}{\bar{G}}_{N}-\;4{\bar{G}}_{N}^{T}\Phi_{N}^{1/2}{\left(\Phi_{N}+4\gamma I_{N,N}\right)}^{-1}{\left(\Phi_{N}+4\gamma I_{N,N}\right)}^{-2}\Phi_{N}^{1/2}{\bar{G}}_{N},$$

which yields (34).

Therefore, using (A17), we obtain

$$\frac{\partial^{2}}{\partial \gamma^{2}}I_{N}^{\gamma }=-8{\bar{G}}_{N}^{T}\Phi_{N}^{1/2}\frac{\partial }{\partial \gamma }{\left(\Phi_{N}+4\gamma I_{N,N}\right)}^{-2}{\left(\Phi_{N}+4\gamma I_{N,N}\right)}^{-1}\Phi_{N}^{1/2}{\bar{G}}_{N}-\;8{\bar{G}}_{N}^{T}\Phi_{N}^{1/2}{\left(\Phi_{N}+4\gamma I_{N,N}\right)}^{-2}\frac{\partial }{\partial \gamma }{\left(\Phi_{N}+4\gamma I_{N,N}\right)}^{-1}\Phi_{N}^{1/2}{\bar{G}}_{N}.$$

Next, applying Formulas (A18) and (A19), we find:

$$\frac{\partial^{2}}{\partial \gamma^{2}}I_{N}^{\gamma }=8\cdot 4{\bar{G}}_{N}^{T}\Phi_{N}^{1/2}{\left(\Phi_{N}+4\gamma I_{N,N}\right)}^{-2}{\left(\Phi_{N}+4\gamma I_{N,N}\right)}^{-2}\Phi_{N}^{1/2}{\bar{G}}_{N}+\;8\cdot 4{\bar{G}}_{N}^{T}\Phi_{N}^{1/2}{\left(\Phi_{N}+4\gamma I_{N,N}\right)}^{-1}{\left(\Phi_{N}+4\gamma I_{N,N}\right)}^{-2}{\left(\Phi_{N}+4\gamma I_{N,N}\right)}^{-1}\Phi_{N}^{1/2}{\bar{G}}_{N}+\;8\cdot 4{\bar{G}}_{N}^{T}\Phi_{N}^{1/2}{\left(\Phi_{N}+4\gamma I_{N,N}\right)}^{-2}{\left(\Phi_{N}+4\gamma I_{N,N}\right)}^{-2}\Phi_{N}^{1/2}{\bar{G}}_{N},$$

whence

$$\frac{\partial^{2}}{\partial \gamma^{2}}I_{N}^{\gamma }=8\cdot 12{\bar{G}}_{N}^{T}\Phi_{N}^{1/2}{\left(\Phi_{N}+4\gamma I_{N,N}\right)}^{-4}\Phi_{N}^{1/2}{\bar{G}}_{N},$$

i.e., Formula (35) follows.

## Appendix B

**Table A1:** For Example I, the square model error $\varepsilon_{N}^{T}\varepsilon_{N}$ (31), the smoothness index $I_{N}^{\gamma }=\int_{0}^{\infty }{\left[{\bar{H}}_{N}^{\gamma }\left(\tau \right)\right]}^{2}d\tau$, Equation (32), and the noise robustness index $Q_{N}^{\gamma }$ (38) for N measurements of the relaxation modulus corrupted by normally distributed additive independent noises with zero mean value and the standard deviation $\sigma =4\left[Pa\right]$; parameter γ is introduced in the modified Lagrange Functional (25).

γ $\left[s^{-1}\right]$	Index	N=20	N=50	N=100	N=500	N=1000
5 × 10^−7^	$\varepsilon_{N}^{T}\varepsilon_{N}$ $\left[{kPa}^{2}\right]$	5.43595 × 10^−4^	1.00519 × 10^−3^	1.84977 × 10^−3^	7.70001 × 10^−3^	1.55419 × 10^−2^
	$I_{N}^{\gamma }$ $\left[{kPa}^{2}\cdot s\right]$	83.336529	70.77716	71.747521	73.185807	83.497189
	$Q_{N}^{\gamma }$ $\left[{kPa}^{2}\cdot s\right]$	6.384582	6.648187	5.829650	5.484982	13.108415
1 × 10^−6^	$\varepsilon_{N}^{T}\varepsilon_{N}$ $\left[{kPa}^{2}\right]$	5.63927 × 10^−4^	1.01733 × 10^−3^	1.85720 × 10^−3^	7.707668 × 10^−3^	1.556163 × 10^−2^
	$I_{N}^{\gamma }$ $\left[{kPa}^{2}\cdot s\right]$	76.168804	66.385898	69.099367	70.438599	76.296796
	$Q_{N}^{\gamma }$ $\left[{kPa}^{2}\cdot s\right]$	3.564357	3.317502	3.031949	2.621451	5.193645
5 × 10^−6^	$\varepsilon_{N}^{T}\varepsilon_{N}$ $\left[{kPa}^{2}\right]$	6.61796 × 10^−4^	1.04504 × 10^−3^	1.887541 × 10^−3^	7.733015 × 10^−3^	1.56079 × 10^−2^
	$I_{N}^{\gamma }$ $\left[{kPa}^{2}\cdot s\right]$	65.608941	63.014540	65.816002	67.562022	70.882789
	$Q_{N}^{\gamma }$ $\left[{kPa}^{2}\cdot s\right]$	0.851793	1.172124	0.914936	0.414335	0.754181
1 × 10^−5^	$\varepsilon_{N}^{T}\varepsilon_{N}$ $\left[{kPa}^{2}\right]$	7.48551 × 10^−4^	1.06649 × 10^−3^	1.92315 × 10^−3^	7.75582 × 10^−3^	1.56467 × 10^−2^
	$I_{N}^{\gamma }$ $\left[{kPa}^{2}\cdot s\right]$	62.549842	62.274208	64.589149	66.768457	69.528702
	$Q_{N}^{\gamma }$ $\left[{kPa}^{2}\cdot s\right]$	0.410221	0.739890	0.596089	0.238231	0.379671

**Table A2:** For Example I, the square model error $\varepsilon_{N}^{T}\varepsilon_{N}$ (31), the smoothness index $I_{N}^{\gamma }=\int_{0}^{\infty }{\left[{\bar{H}}_{N}^{\gamma }\left(\tau \right)\right]}^{2}d\tau$, Equation (32), and the noise robustness index $Q_{N}^{\gamma }$ (38) for N measurements of the relaxation modulus corrupted by normally distributed additive independent noises with zero mean value and the standard deviation $\sigma =6\left[Pa\right]$; parameter γ is introduced in the modified Lagrange functional (25).

γ $\left[s^{-1}\right]$	Index	N=20	N=50	N=100	N=500	N=1000
5 × 10^−7^	$\varepsilon_{N}^{T}\varepsilon_{N}$ $\left[{kPa}^{2}\right]$	1.21470 × 10^−3^	2.25864 × 10^−3^	4.16166 × 10^−3^	1.73258 × 10^−2^	3.49726 × 10^−2^
	$I_{N}^{\gamma }$ $\left[{kPa}^{2}\cdot s\right]$	96.874904	77.140666	77.6860944	79.138260	100.199781
	$Q_{N}^{\gamma }$ $\left[{kPa}^{2}\cdot s\right]$	14.365310	14.958420	13.116713	12.341209	29.493933
1 × 10^−6^	$\varepsilon_{N}^{T}\varepsilon_{N}$ $\left[{kPa}^{2}\right]$	1.24932 × 10^−3^	2.28259 × 10^−3^	4.17777 × 10^−3^	1.73428 × 10^−2^	3.50181 × 10^−2^
	$I_{N}^{\gamma }$ $\left[{kPa}^{2}\cdot s\right]$	84.636611	68.436067	71.912153	73.025494	83.586342
	$Q_{N}^{\gamma }$ $\left[{kPa}^{2}\cdot s\right]$	8.019804	7.464379	6.821885	5.898264	11.685702
5 × 10^−6^	$\varepsilon_{N}^{T}\varepsilon_{N}$ $\left[{kPa}^{2}\right]$	1.39704 × 10^−3^	2.32565 × 10^−3^	4.22678 × 10^−3^	1.73873 × 10^−2^	3.51075 × 10^−2^
	$I_{N}^{\gamma }$ $\left[{kPa}^{2}\cdot s\right]$	68.346247	62.895476	66.290649	67.641705	72.583587
	$Q_{N}^{\gamma }$ $\left[{kPa}^{2}\cdot s\right]$	1.916535	2.637279	2.058606	0.932255	1.696907
1 × 10^−5^	$\varepsilon_{N}^{T}\varepsilon_{N}$ $\left[{kPa}^{2}\right]$	1.51741 × 10^−3^	2.34999 × 10^−3^	4.27376 × 10^−3^	1.74152 × 10^−2^	3.516484 × 10^−2^
	$I_{N}^{\gamma }$ $\left[{kPa}^{2}\cdot s\right]$	64.090823	62.048757	64.664280	66.661361	70.566065
	$Q_{N}^{\gamma }$ $\left[{kPa}^{2}\cdot s\right]$	0.922997	1.664754	1.341202	0.536019	0.854259
